# Oxidative Stress in Mammalian Cells Impinges on the Cysteines Redox State of Human XRCC3 Protein and on Its Cellular Localization

**DOI:** 10.1371/journal.pone.0075751

**Published:** 2013-10-08

**Authors:** Pierre-Marie Girard, Dany Graindorge, Violetta Smirnova, Pascal Rigolet, Stefania Francesconi, Susan Scanlon, Evelyne Sage

**Affiliations:** 1 Institut Curie, Centre de Recherche, Orsay, France; 2 CNRS, UMR3348, Orsay, France; 3 Université Paris-Sud 11, Orsay, France; University of California Merced, United States of America

## Abstract

In vertebrates, XRCC3 is one of the five Rad51 paralogs that plays a central role in homologous recombination (HR), a key pathway for maintaining genomic stability. While investigating the potential role of human XRCC3 (hXRCC3) in the inhibition of DNA replication induced by UVA radiation, we discovered that hXRCC3 cysteine residues are oxidized following photosensitization by UVA. Our *in silico* prediction of the hXRCC3 structure suggests that 6 out of 8 cysteines are potentially accessible to the solvent and therefore potentially exposed to ROS attack. By non-reducing SDS-PAGE we show that many different oxidants induce hXRCC3 oxidation that is monitored in Chinese hamster ovarian (CHO) cells by increased electrophoretic mobility of the protein and in human cells by a slight decrease of its immunodetection. In both cell types, hXRCC3 oxidation was reversed in few minutes by cellular reducing systems. Depletion of intracellular glutathione prevents hXRCC3 oxidation only after UVA exposure though depending on the type of photosensitizer. In addition, we show that hXRCC3 expressed in CHO cells localizes both in the cytoplasm and in the nucleus. Mutating all hXRCC3 cysteines to serines (XR3/S protein) does not affect the subcellular localization of the protein even after exposure to camptothecin (CPT), which typically induces DNA damages that require HR to be repaired. However, cells expressing mutated XR3/S protein are sensitive to CPT, thus highlighting a defect of the mutant protein in HR. In marked contrast to CPT treatment, oxidative stress induces relocalization at the chromatin fraction of both wild-type and mutated protein, even though survival is not affected. Collectively, our results demonstrate that the DNA repair protein hXRCC3 is a target of ROS induced by environmental factors and raise the possibility that the redox environment might participate in regulating the HR pathway.

## Introduction

Reactive oxygen species (ROS) are produced endogenously as oxidative by-products of mitochondria metabolism or in response to a wide range of environmental factors such as ionizing radiation (IR), ultraviolet (UV) radiation, air pollutants, pesticides or pharmaceutical drugs. UVA radiation (320–400 nm), the predominant UV component of sunlight reaching the Earth’s surface, causes a range of damage to cellular biomolecules [1], including direct photo-induced damage to protein [2] and to DNA [3]. However, the primary cytotoxic effects of UVA are due to ROS, especially singlet oxygen (^1^O_2_) that is generated from the interaction of photons with intracellular [4] and/or extracellular [5] photosensitizers, and transfer of energy to molecular oxygen, converting it from its triplet ground state (^3^O_2_) to a highly reactive singlet state (^1^O_2_) [6]. In turn, ^1^O_2_ causes oxidative damage to proteins, DNA and lipids [2], [3], [7]. The rapid inactivation of ^1^O_2_ in aqueous solution led to the idea that the primary reactions of ^1^O_2_ in cells would be localized at the site of ^1^O_2_ formation [8], [9].

Proteins are major targets for ^1^O_2_, with damage occurring preferentially at Trp, His, Tyr, Met, and Cys side-chains [10]. For example, Maresca et al. have shown that ^1^O_2_ produced by UVA is able to modify the charge properties of catalase and that this could imply oxidative modifications of Trp and Met residues [11]. ^1^O_2_ also causes covalent oxidative crosslinking between the Proliferating Cell Nuclear Antigen (PCNA) subunits, likely due to a histidine-lysine crosslinking [12], and inactivation of protein tyrosine phosphatase-1B (PTP1) by oxidation of the active site cysteine [13]. In fact, the thiol function in cysteine residues is among the most susceptible one and can undergo several oxidation states. The sulfhydryl group (-SH) of cysteine can be sequentially oxidized to sulfenic acid (-SOH), a key intermediate in the formation of intra- and inter-chain disulfide bonds (-S-S-), to sulfinic (-SO_2_H) or to sulfonic (-SO_3_H) acid (see for review [14]). Unlike sulfenic acids that can be reduced by major cellular reductants, sulfinic and sulfonic acids cannot. Oxidation of Cys residues in proteins can lead to diverse functional consequences, such as inhibition or activation of enzymatic activities, inhibition of binding activities [13], [15]–[16]. To maintain the intracellular thiol-disulfide redox status under reducing conditions ([-SH]**>**[-S-S-]), living cells possess two major systems, the thioredoxin (Trx) and glutaredoxin (Grx) pathways [17]. All members of the Trx and Grx families, with the exception of Grx5, catalyse the reversible reduction of disulfides by a thiol-dependent thiol-disulfide exchange reaction [18]. Although Trxs are the main thiol-disulfide oxidoreductases that catalyse the reduction of disulfide bonds in many proteins, Grxs specifically and efficiently promote protein-SSG de-glutathionylation [19], [20].

Glutathione (GSH) is a water-soluble tripeptide consisting of glycine, cysteine, and glutamic acid (L-glutamyl-L-cysteinylglycine) with essential roles as an antioxidant and intracellular redox buffer. In mammalian cells, it is the most abundant low molecular mass thiol (1 to 10 mM), localizing predominantly in the cytosol (90%) and present at 98% in the thiol-reduced form (GSH/GSSG ratio ≈ 100/1). Upon severe oxidative stress, the GSH/GSSG ratio can drastically shift (see for reviews [21], [22]). S-glutathionylation, which is the formation of mixed disulfides between protein thiols and GSH (prot-SSG), has gained particular attention over the last few years as a potential mechanism for the post-translational regulation of a variety of proteins, in both normal and pathophysiological processes [23], [24], and for protecting sensitive protein thiols from irreversible oxidation [25].

In vertebrates, XRCC3 is one of the five RAD51 paralogs (*i.e.* XRCC2, XRCC3, RAD51L1/RAD51B, RAD51L2/RAD51C and RAD51L3/RAD51D) that functions in the repair of DNA double strand breaks by homologous recombination (HR) [26]. In cells, XRCC3 interacts with its binding partner Rad51C [27], [28]. XRCC3 deficiency results in impaired DNA damage-induced RAD51 foci formation, impaired HR, elevated chromosome aberrations and increased sensitivity to camptothecin (CPT) and many DNA cross-linking agents [29], [30]. Human XRCC3 (hXRCC3), which contains 8 cysteine residues, was identified from a cosmid library through its ability to complement the mutagen-sensitive CHO line irs1SF [30].

Biverstål et al. reported that UVA radiation might induce a complex type of lesion that blocks fork progression and requires HR to be repaired [31]. On the other hand, fork slowing on damaged vertebrate chromosomes is an active process that requires the HR protein XRCC3 [32]. As we have previously reported that UVA-induced ROS impinged on DNA replication through a mechanism that does not require functional DNA integrity checkpoint pathways [33], [34], we thought to investigate the role of HR in ROS-induced slow down of DNA replication using HR proficient and deficient cells. During our investigations, we discovered that cysteine residues of hXRCC3 are oxidized by UVA-induced ROS. This finding prompted us to better characterize the redox state of hXRCC3 according to the cell type, the nature of the oxidant and the intracellular content of GSH.

## Results

### XRCC3 is Dispensable for the UVA-induced Inhibition of DNA Synthesis and Cytotoxicity

**Figure 1:**
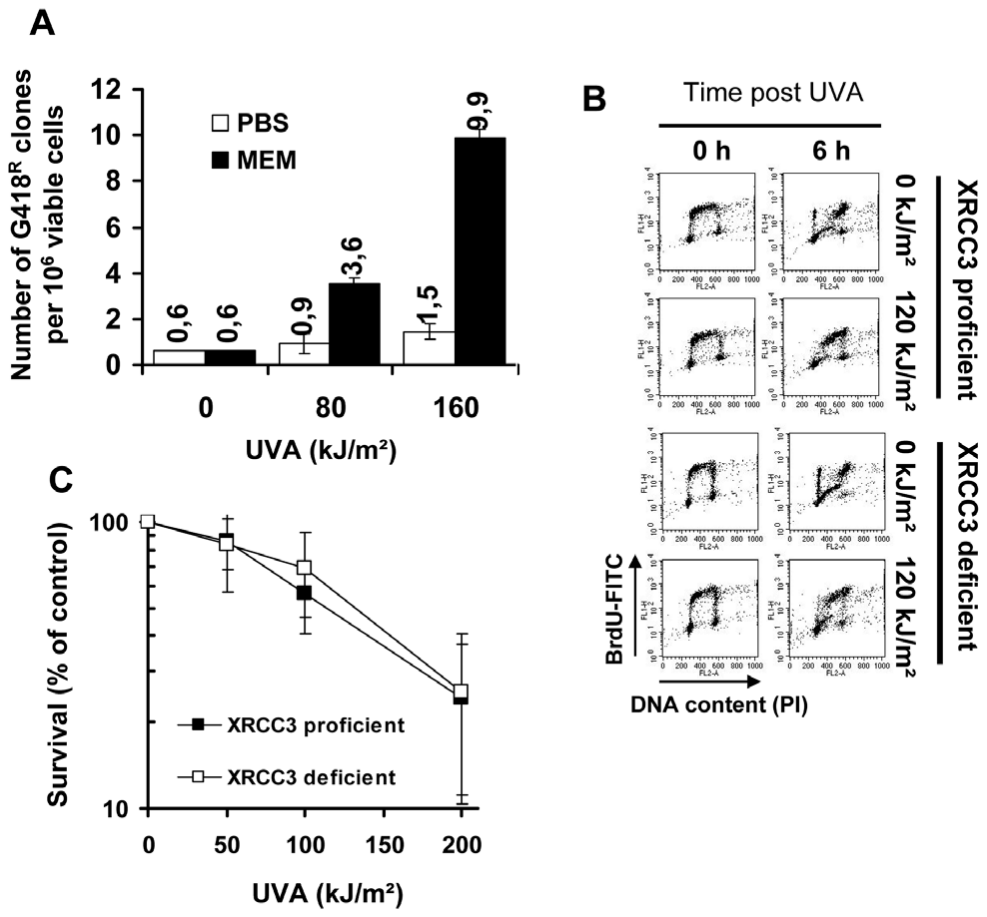
Dispensable role of hXRCC3 in the delay of S-phase induced by UVA_MEMi_ and in protecting cells from UVA phototoxicity. (**A**) CHO DRA10 cells were exposed to increasing doses of UVA in MEMi or PBS and the number of G418^R^ clones scored after selection. Results are the mean ± SD of three independent experiments. (**B**) XRCC3 proficient (CXR3) and deficient (irs1SF) CHO cells were labeled with BrdU, irradiated at 120 kJ/m^2^ UVA_MEMi_ and trypsinized after 6 hours of recovery. Cells were fixed in 70% EtOH and analysed by flow cytometry for BrdU incorporation (FITC-BrdU) and DNA content (PI). The data shown are representative of two independent experiments. (**C**) XRCC3 proficient and deficient cells were exposed to increasing doses of UVA_MEMi_, trypsinized, and plated at low density. The colonies were stained after 8–10 days incubation at 37°C. Results are the mean ± SD of three independent experiments.

We have previously reported that exposure of mammalian cells to UVA radiation in serum- and phenol red-free medium (UVA_MEMi_), but not in PBS (UVA_PBS_), led to a strong inhibition of DNA synthesis [34]. This medium contains vitamins among which a potent photosensitizer, riboflavin [4]. To investigate the putative role of HR in this process, we asked if UVA radiation stimulates HR. To do so, we used CHO DRA10 cells that allow the selection of G418 resistant clones resulting from repair by HR of an intrachromosomal recombination substrate [35], [36]. Therefore, cells were exposed to 0, 80 and 160 kJ/m^2^ of UVA_PBS_ or UVA_MEMi_, and grown on G418 selective medium. If compared to mock cells, cells irradiated at 160 kJ/m^2^ in PBS or MEMi displayed a 2.5 and 16.5 fold increase in HR, respectively (Figure 1A). Thus, the presence of a photosensitizer during UVA radiation strongly stimulates HR, likely due to the ROS [34].

Next, we used XRCC3 proficient (CXR3) and deficient (irs1SF) CHO cells to investigate the role of XRCC3 in UVA-induced S-phase delay. Cells were pulse-labeled with BrdU, exposed to 120 kJ/m^2^ of UVA_MEMi_ and allowed to recover for 6 h. Analysis of the samples by flow cytometry revealed that S-phase was slowed down by UVA_MEMi_ in XRCC3 deficient cells (Figure 1B). We confirmed these results using human cells in which XRCC3 expression was down regulated by siRNA or by gene disruption (Supplemental Figure S1). Furthermore, we showed that XRCC3 is not required to protect cells from the toxic effects of UVA radiation (Figure 1C). Together, these data indicate that, despite its stimulation, HR pathway does not contribute to inhibition of DNA synthesis nor to cell survival in response to UVA-induced ROS.

### Photosensitization by UVA Induces the Reversible Oxidation of hXRCC3

**Figure 2:**
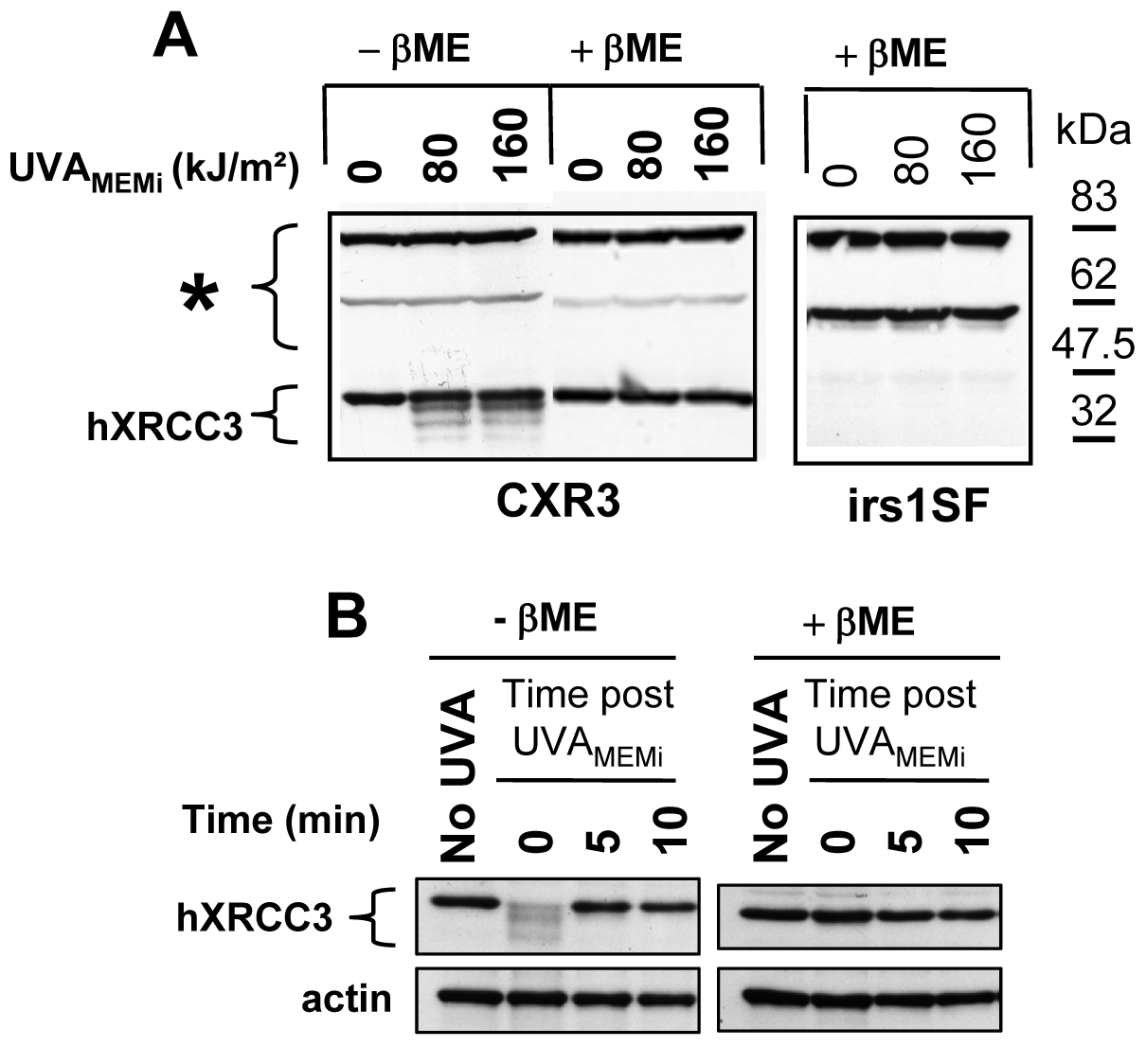
Reversible oxidation of hXRCC3. CXR3 cells were exposed to different doses of UVA_MEMi_ and total soluble protein extracts were prepared either immediately (**A**) or at various times post radiation (**B**). irs1SF cells were used as negative control for XRCC3 expression. Cells were lysed on ice in lysis buffer containing 10 mM N-ethylmaleimide (NEM). Protein extracts in non reductive (-ßME) and reductive (+ßME) conditions were loaded on 9% SDS-PAGE and transferred onto nitrocellulose membrane. The membranes were probed with rabbit polyclonal anti-XRCC3 antibody. (*) indicates non-specific cross-reactivity of the antibody. Actin was used as loading control. ßME: ß-mercaptoethanol.

As we hypothesized that protein oxidation contributes to a certain extent to the overall harmful effects of UVA radiation [34], we asked if XRCC3 could be a target of UVA-induced ROS. To check this hypothesis, the CHO cell line CXR3, which expresses human XRCC3 [30], was exposed to 0, 80 and 160 kJ/m^2^ UVA_MEMi_ and soluble protein extracts were prepared immediately post radiation in the presence of N-ethylmaleimide (NEM), an irreversible thiol alkylating agent that protects cysteine residues from oxidation during cell lysis, acetone precipitation and/or electrophoresis. Human XRCC3 protein, whose apparent molecular mass is around 37 kDa (Swiss-Prot #: O43542), was detected by Western blot using an antibody directed against the C-terminal part of hXRCC3 encompassing Cys328 (amino acids 315 to 346, Novus Biologicals, personal communication). Specificity of the antibody was confirmed by looking at hXRCC3 expression in XRCC3 proficient (siCtr-transfected MRC5Vi, HCT116 W.T. and CXR3) and deficient (siXRCC3-transfected MRC5Vi, HCT116 XRCC3^−/−^and irs1SF) cells (Figure 2A and Figures S1A and S1B).

We observed changes in the electrophoretic mobility of hXRCC3 towards lower molecular weights (Figure 2A, condition −ßME) that might correspond to intramolecular disulfides. Indeed, the formation of intramolecular disulfides is predicted to increase SDS-PAGE mobility because of a decrease in the hydrodynamic radius of the SDS-bound polypeptide, especially if the two constitutive Cys residues are far apart in the primary sequence. Noteworthy, these changes were completely reversed *in vitro* by a reducing agent (Figure 2A, condition +ßME), as well as in cells at 5 min after irradiation (Figure 2B). To confirm these observations in human cells, similar experiments were conducted in MRC5Vi, HaCaT and HCT116 cells. Surprisingly, we did not observe a change in the electrophoretic mobility of the protein but rather a slight decrease of its immunodetection in response to photosensitization by UVA_MEMi_ (Figure S2A). These changes were also reversed *in vitro* by a reducing agent and intracellularly at 5 to 10 min after irradiation (Figures S2B and S2C). These results indicate that photosensitization by UVA_MEMi_ induces the oxidation of cysteine residues of hXRCC3, likely by the formation of one or more intramolecular disulfide bonds that are rapidly reduced in cells.

### Cysteines 86, 141, 193, 221, 310 and 328 of Human XRCC3 are Potentially Accessible to the Solvent

**Figure 3:**
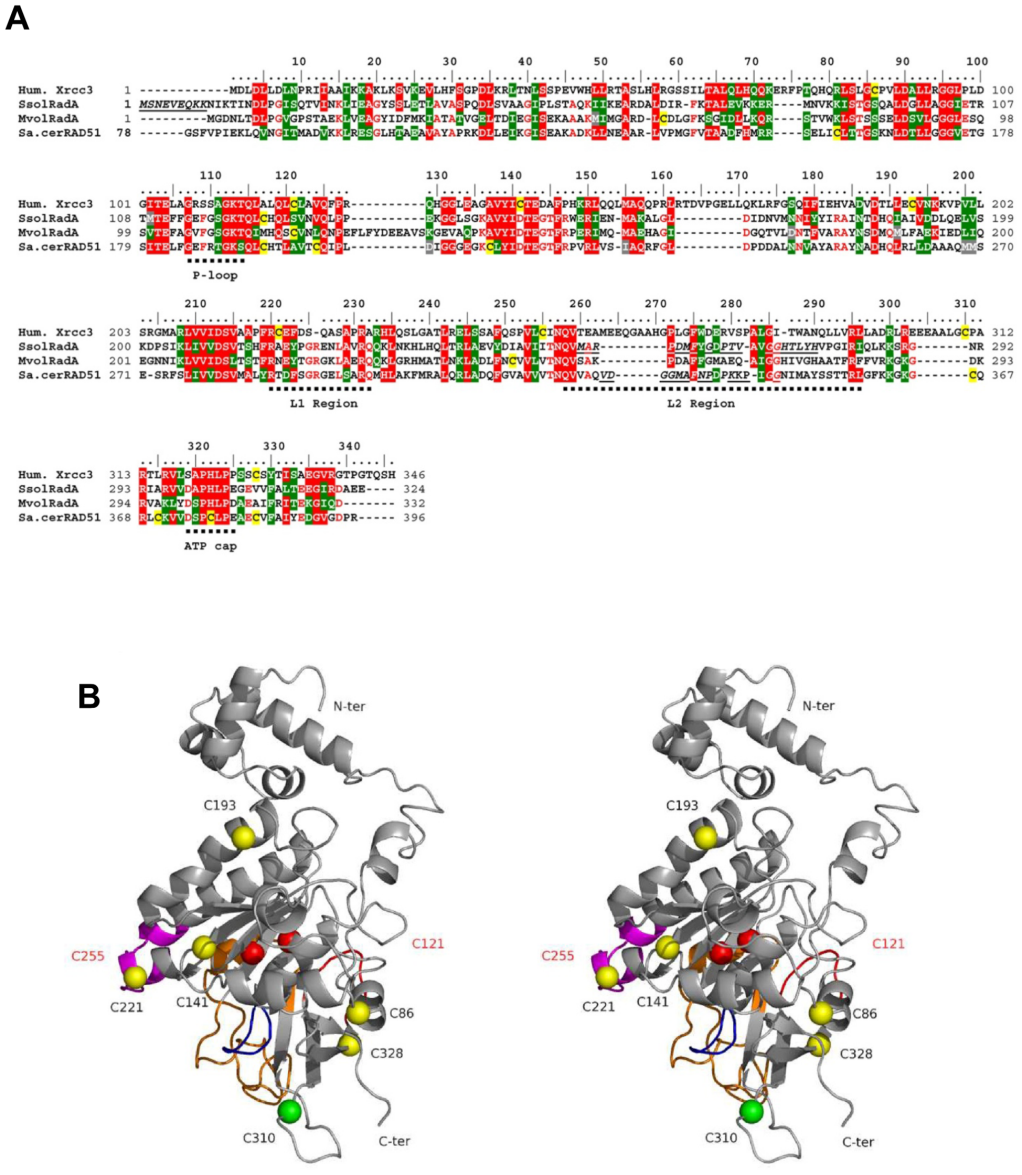
Spatial cysteine distribution in human XRCC3. (A) Sequence alignment of the archaeal RadA from *Sulfolobus solfataricus*, the archaeal RadA from *Methanococcus voltae*, the RAD51 protein of *Saccharomyces cerevisiae* and the human XRCC3 (Hum. Xrcc3) performed with the program ClustalW. These enzymes share characteristic features like the L1 and L2 DNA putative binding regions, an ATP cap, a P-loop as well as conserved residues involved in ATP binding or ATPase activity. Conserved amino acid residues are shadowed in red. The positions of cysteine residues in the four structures are shadowed in yellow. The missing residues in the template crystal structures are underlined and in italic. (**B**) Stereo ribbon diagram showing the positions of the 8 cysteine residues on the modeled structure of the human XRCC3. The Cα positions of Cys86, Cys141, Cys193, Cys221, and Cys328, potentially solvent accessible, are figured in yellow. In red are the positions of Cys121 and Cys255, both buried in the structure of XRCC3. The Cα position of residue Cys310 is in green. Panels B was made with Pymol molecular visualisation system (DeLano Scientific LLC).

To date, the three-dimensional structure of human XRCC3 has not been solved. In order to evaluate the accessibility of the sulfhydryl groups of hXRCC3 to ROS, we built a model of hXRCC3 generating a structure for all the regions of the enzyme. This model is based on the crystal structures of the RAD51 protein of *Saccharomyces cerevisiae* and of the archaeal RadA from *Sulfolobus solfataricus* and from *Methanococcus voltae*, which display around 30 percent sequence identity with the human XRCC3 (Figure 3A). The modeled structure suggests that 6 out of the 8 cysteine residues of human XRCC3 (Cys86, Cys141, Cys193, Cys221, Cys310 and Cys328) are potentially accessible to the solvent, meaning that they are susceptible to changes of the intracellular redox environment (Figure 3B). Furthermore, it appears from this model that the closest C_α_–C_α_ distance among these cysteine residues, which is 9.5 Å between Cys86 and Cys328, is greater than the canonical values of 4 to 7.5 Å observed in cysteine disulfide bridges [37].

### Cys86 and Cys328 are Dispensable for Cell Survival in Response to Camptothecin

As we observed oxido-reduction of hXRCC3 thiols in response to oxidative stress, we asked if sulfhydryl groups are essential for hXRCC3 activity by mutating all cysteines to serines (XR3/S) in myc-tagged hXRCC3 (XR3/C) vector. Furthermore, based on our *in silico* model, we aimed to test the possibility that Cys86 and Cys328 form a disulfide bridge after oxidative stress. Therefore, Cys86 or Cys328 were also independently mutated to Ser (XR3/S86 and XR3/S328). Each construct was stably transfected into irs1SF cells and independent clones were used to study the oxidation of hXRCC3 in response to UVA_MEMi_ and the sensitivity of cells to camptothecin (CPT) in order to assess the efficiency of DNA repair by HR [38], [39].

**Figure 4:**
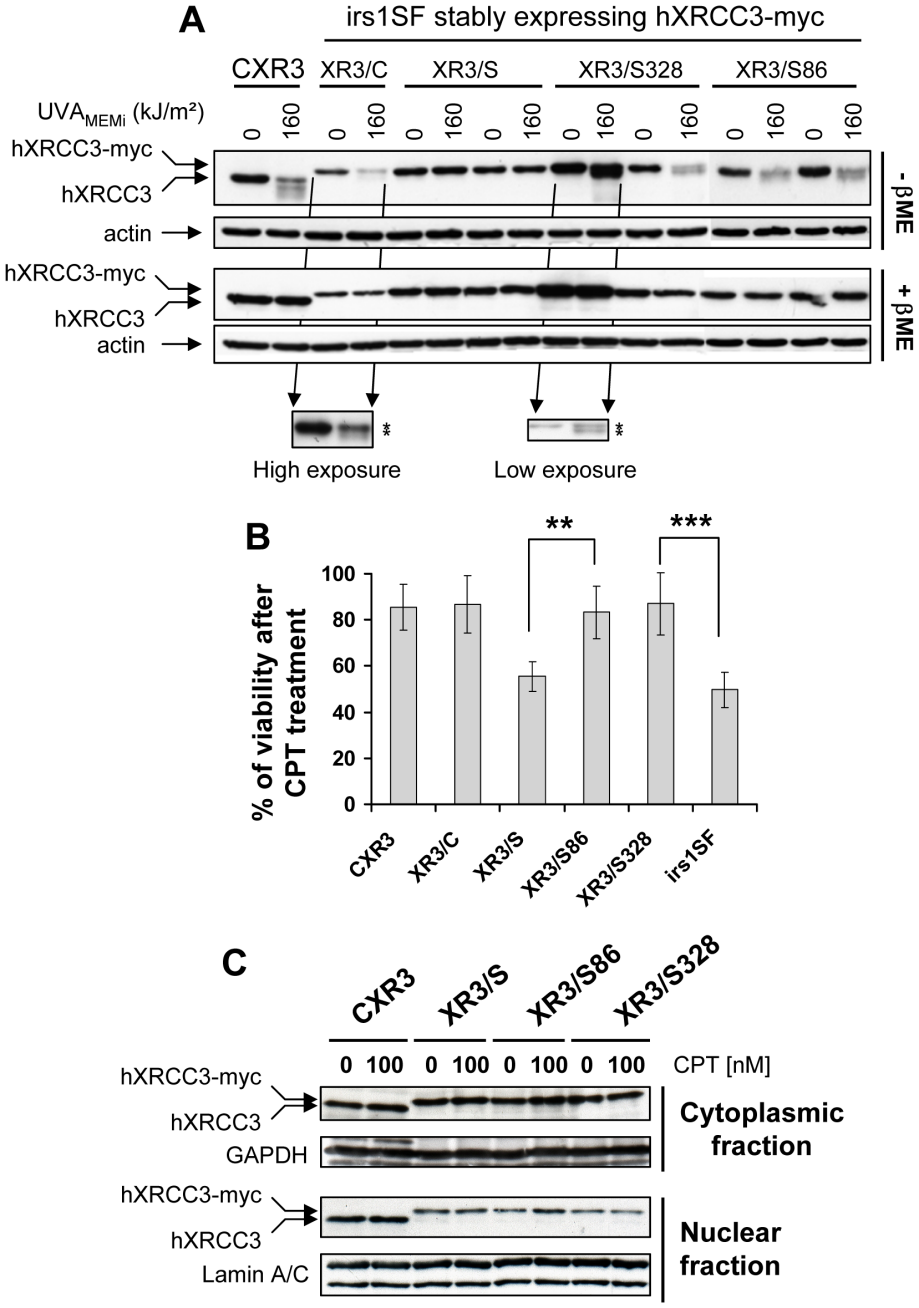
Cys86 and Cys328 are dispensable for hXRCC3 activity. XRCC3-deficient cells (irs1SF) were stably transfected with empty vector or vectors bearing wild-type hXRCC3 (XR3/C), hXRCC3 mutated at all cysteines (XR3/S), at cysteine 86 (XR3/S86) or at cysteine 328 (XR3/S328). (**A**) Cells were exposed to 160 kJ/m^2^ UVA_MEMi_ and immediately lysed post radiation in lysis buffer containing 10 mM NEM. hXRCC3 and hXRCC3-myc tagged proteins were analysed by Western blot using anti-XRCC3 antibody. Actin detection was used as loading control. ßME: ß-mercaptoethanol. Two independent clones were analysed for each mutant protein (XR3/S, XR3/S328 and XR3/S86). (**B**) Cells were exposed to the DNA damaging agent CPT for 16 h and viability was assessed 48 h post treatment by MTT assay. Results are the mean ± SD of 3 to 8 independent experiments using 2 independent clones for each cell line. Statistical analysis was performed using ANOVA with TUKEY’s post test. **P<0.01; ***P<0.001. We indicate only the significant statistical differences relevant for discussion. (**C**) The cytoplasmic and nuclear distributions of hXRCC3 were analysed in untreated and CPT-treated cells. GAPDH and Lamin A/C were used as loading control for the cytoplasmic and nuclear fraction, respectively. The blots are representative of two independent experiments.

Firstly, cells expressing either the wild-type proteins (CXR3 and XR3/C) or the mutated proteins (XR3/S86, XR3/S328 and XR3/S) were exposed to UVA_MEMi_ and the electrophoretic mobility of hXRCC3 was analysed by Western blot. hXRCC3 oxidation was completely abolished in cells expressing XR3/S mutant protein (Figure 4A), thus confirming that the change of hXRCC3 mobility is indeed due to cysteine oxidation. In the others cell lines (CXR3, XR3/C, XR3/S86 and XR3/S328), the electrophoretic mobility of the protein after UVA_MEMi_ revealed the presence of two bands (Figure 4A). Though these experiments did not demonstrate the formation of a disulfide bridge between Cys86 and Cys328, they show that cysteines other than Cys86 and Cys328 can form disulfide bond(s) in response to UVA_MEMi_.

Next, the activity of the wild-type and mutated hXRCC3-myc proteins in cells was estimated by measuring the cell viability in response to CPT, and by comparing it to the viability of the two parental cell lines, CXR3 and irs1SF. We found that XR3/S86 and XR3/S328 cells are no more sensitive to 10 nM CPT than CXR3 and XR3/C cells (Figure 4B). In contrast, cells lacking hXRCC3 (irs1SF) or bearing hXRCC3 mutated at all cysteine residues (XR3/S) exhibit almost a two-fold increase in the sensitivity to 10 nM CPT if compared to CXR3 or XR3/C cells (Figure 4B). We checked the subcellular localization of hXRCC3 in the different cell lines and found that all the proteins localize both in the cytoplasm and the nucleus before and after CPT treatment (Figure 4C). Therefore, cysteine residues of hXRCC3 are not essential to ensure proper localization of the protein to the nucleus, but some, others than Cys86 and Cys328, are essential to allow an efficient HR-dependent DNA repair.

### hXRCC3 Oxidation is due to ^1^O_2_ Generated by UVA_MEMi_

**Figure 5:**
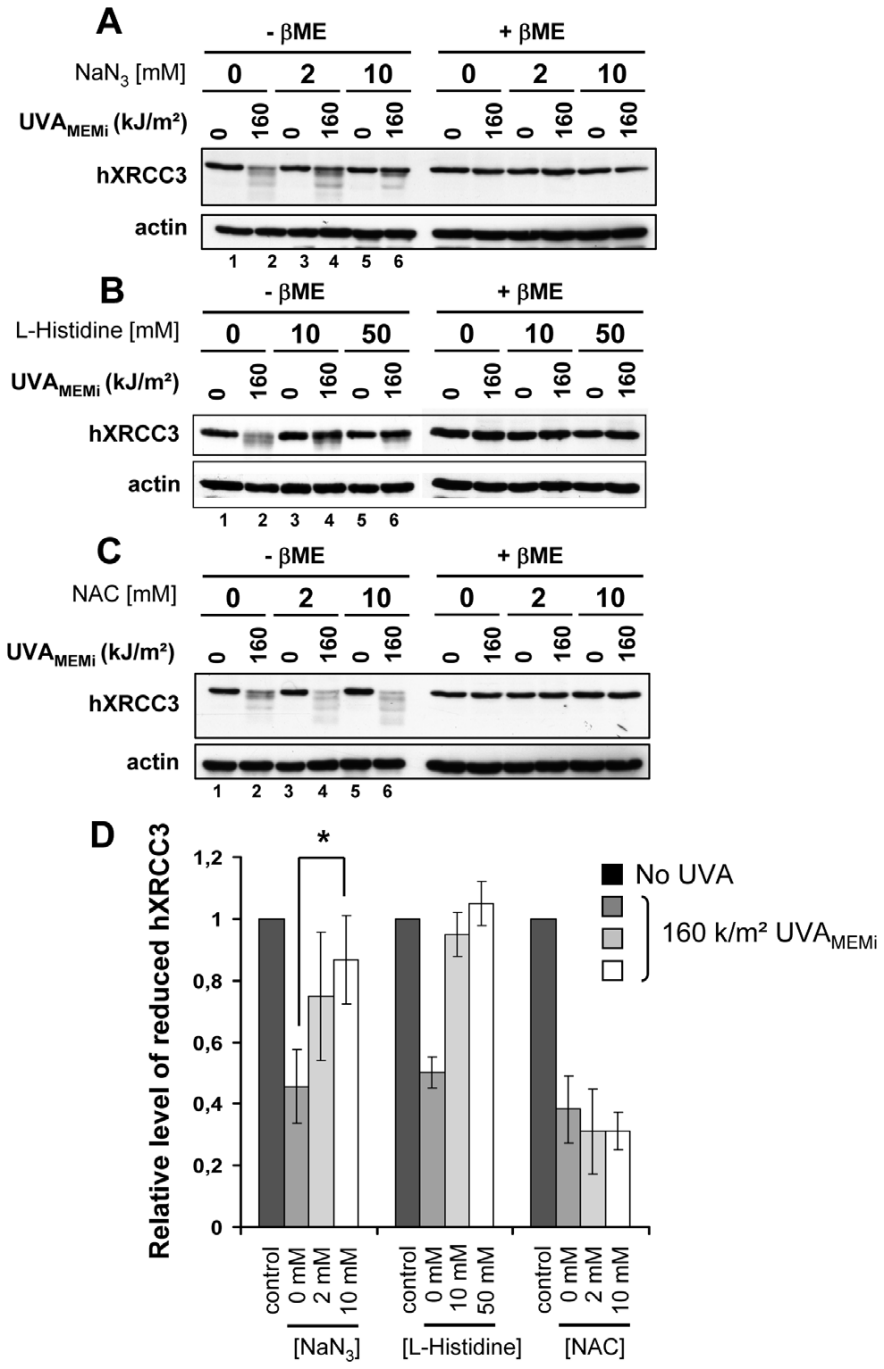
Oxidation of hXRCC3 by UVA_MEMi_ is prevented by NaN_3_ and L-Histidine but not by NAC. CXR3 cells were irradiated in MEMi at 160/m^2^ UVA in the presence of increasing concentration of NaN_3_ (**A**), L-Histidine (**B**) or NAC (**C**). The blots shown are representative of 4 independent experiments for (A) and 2 for (B) and (C). XRCC3 and actin were analysed by Western blot. ßME: ß-mercaptoethanol. (D) The relative level of reduced hXRCC3 in conditions (A), (B) and (C) was calculated by dividing the intensity of the top band in UVA-treated cells (lanes 2,4 and 6) by the intensity of the same band in the appropriate unirradiated cells (lanes 1, 3 and 5). Statistical analysis for the NaN_3_ treatment was performed using ANOVA with TUKEY’s post test. Only the significant statistical difference relevant for discussion is shown. * P<0.05.

Because photosensitization by UVA is known to generate mainly ^1^O_2_ [4], we examined its implication in hXRCC3 oxidation by irradiating CHO cells in the presence of increasing concentrations of NaN_3_ (Figure 5A) or L-Histidine (Figure 5B), two quenchers of ^1^O_2_ [40], [41], or of N-acetyl-L-cysteine (NAC) (Figure 5C) that scavenges free radicals [42]. Following quantifications, we found that hXRCC3 oxidation by UVA radiation was significantly prevented by increasing the concentration of NaN_3_ or L-Histidine but not of NAC (Figure 5D). Similar results were obtained with the human cell line MRC5Vi (Figure S3). These data point to ^1^O_2_ as the main UVA-induced ROS that leads to the formation of oxidized cysteines in hXRCC3.

### The Intracellular Pool of GSH/GSSG is Required for the Oxidation of hXRCC3 by UVA_MEMi_

**Figure 6:**
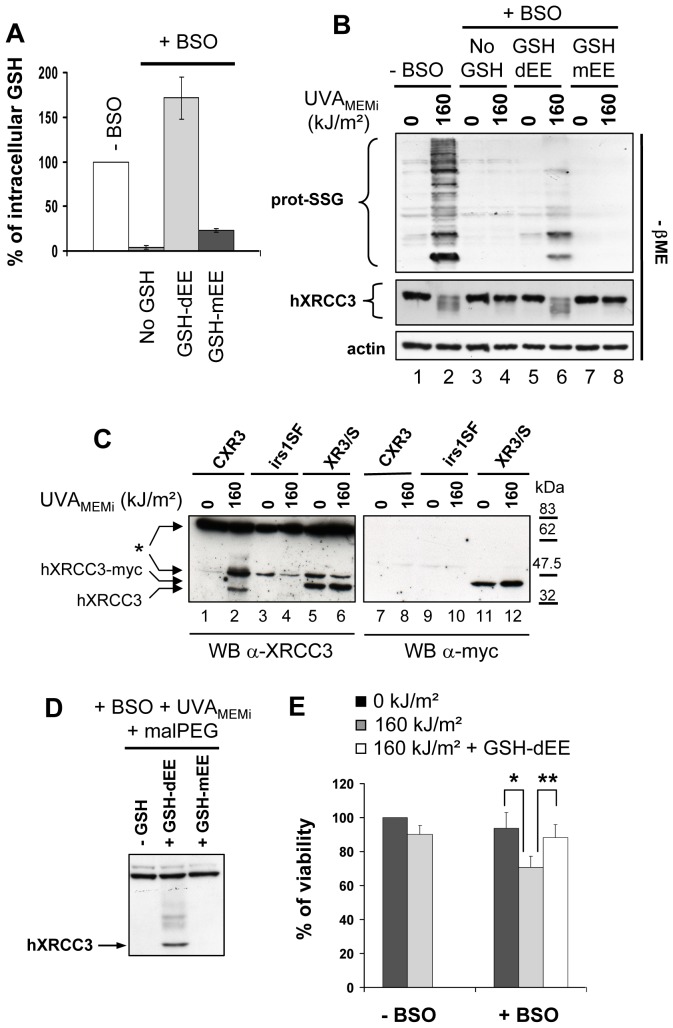
Depletion of intracellular GSH/GSSG pool prevents hXRCC3 oxidation induced by UVA_MEMi_. CXR3 cells were pre-incubated for 24 h in culture medium containing or not 0.5 mM BSO. Thereafter, BSO-treated cells were complemented or not with 2 mM GSH-dEE or GSH-mEE for 1 h. (**A**) Measurement of GSH level in cells. Values are expressed as % of GSH relative to control cells (–BSO) and results are the mean ± SD of 3 independent experiments. Cells treated as described in panel A were exposed to 160 kJ/m^2^ UVA_MEMi_ and lysed immediately post radiation in buffer containing either 10 mM NEM (**B**) or 4 mM malPEG (**D**). (**C**) CXR3, irs1SF and XR3/S cells were exposed to 160 kJ/m^2^ UVA_MEMi_ and immediately lysed in buffer containing 4 mM malPEG. The star (*) indicates non specific cross-reactivity of anti-XRCC3 antibody. In (C) and (D), the samples were analysed in reducing condition (+ßME). Myc-tagged XR3/S protein was detected using anti-myc antibody, and S-glutathionylated proteins using anti-GSH antibody. Actin was used as loading control. ßME: ß-mercaptoethanol. (**E**) GSH-proficient (−BSO and +BSO +GSH-dEE) and deficient (+BSO) cells were irradiated at 160 kJ/m^2^ UVA_MEMi_ and cell viability was assessed 24 h post treatment by MTT assay. Values are the mean +/− SD of 5 independent experiments. Statistical analysis was performed using ANOVA with TUKEY’s post test. * P<0.05; ** P<0.01.

As NAC, a GSH precursor [43], seemed to favour hXRCC3 oxidation by UVA_MEMi_ (Figure 5C), we postulated that the endogenous pool of GSH/GSSG might participate in hXRCC3 oxidation. At first we used DL-buthionine-[*S*,*R*]-sulfoximine (BSO), an inhibitor of GSH biosynthesis, to deplete the intracellular GSH/GSSG pool [44], [45]. Thereafter, glutathione di-ethylester (GSH-dEE) or mono-ethylester (GSH-mEE) was used to modulate the intracellular GSH level in BSO-treated cells. BSO treatment resulted in ∼ 90% depletion of the intracellular GSH/GSSG pool (Figure 6A) without loss of viability (Figure S4A). In agreement with previously published data [46], [47], we found that only GSH-dEE, but not GSH-mEE, was able to restore the intracellular level of GSH in BSO-treated cells (Figure 6A). Normal and GSH-depleted cells were exposed to UVA_MEMi_, and both hXRCC3 oxidation and protein S-glutathionylation were analysed by Western blot. As shown in Figure 6B, the formation of disulfides in hXRCC3 and of protein-SSG adducts by the oxidative stress was detected only in GSH-proficient cells (lanes 2 and 6) but not in GSH-deficient cells (lanes 4 and 8). By checking in the cells the formation of UVA_MEMi_-induced ROS and the covalent oxidative crosslinking of PCNA (Proliferating Cell Nuclear Antigen) subunits, which is a biomarker of photodynamic damage due to ^1^O_2_ [12], [41], we excluded the possibility that BSO treatment could prevent the oxidation of proteins in response to photosensitization by UVA_MEMi_ (Supplemental Figures S4B and S4C).

To further characterize the redox state of hXRCC3 cysteine residues after UVA_MEMi_, we used methoxypolyethylene glycol 5,000 maleimide (malPEG) in the lysis buffer instead of NEM. Indeed, malPEG is a specific and irreversible thiol alkylator that reacts with -SH groups exposed to the solvent, leading to an increase of the apparent molecular mass of the protein of 5 kDa per alkylated SH. In contrast, oxidized thiols cannot react with malPEG. Cells lacking hXRCC3 (irs1SF) or expressing either hXRCC3 protein (CXR3) or the myc-tagged XR3/S proteins were exposed to UVA_MEMi_ and immediately lysed in the presence of malPEG. Using anti-XRCC3 antibody, we observed one, two or three bands in each lane (Figure 6C). The bands around 83 and 47 kDa (marked by an asterix) were also observed in irs1SF cells (lanes 3 and 4) and correspond to unspecific immunodetections. The band between the 47.5 and the 32 kDa protein markers corresponds to untagged hXRCC3 (lane 2) and myc-tagged XR3/S protein (lanes 5 and 6), which is confirmed by blotting the membranes with anti-myc antibody (Figure 6C, lanes 7 to 12).

In unirradiated CXR3 cells, we failed to see hXRCC3 alone or conjugated to malPEG molecules (Figure 6C, lane 1) while NEM-conjugated hXRCC3 proteins were immunodetected (Figures S5A and S5B). In contrast, XR3/S protein that can not react with malPEG was immunodetected in unirradiated and irradiated cells (Figure 6C, lanes 5 and 6). These data indicate that Cys328 is likey to be exposed to the solvent in the structure of hXRCC3 and that addition of a bulky adduct (malPEG) to Cys328 of hXRCC3 prevents its immunodetection. Such inhibitory effect on the immunodetection of malPEG–protein conjugates was also observed when the membranes were probed with anti-PCNA or anti-GAPDH antibodies (Figures S5C and S5D).

Following exposure to UVA_MEMi_, the presence of a band that migrates at the expected position of hXRCC3 protein (∼ 38 kDa) (Figure 6C, lane 2), revealed that all accessible thiols of hXRCC3, including Cys328, were oxidized after UVA_MEMi_ in CXR3 cells. By loading on the same gel (SDS-PAGE in reducing conditions) total soluble protein extracts from NEM or malPEG-treated cells followed by quantification of the intensity of the full length protein, we found that approximately 25 to 30% of the total amount of protein is oxidized at all accessible cysteines. Using malPEG in the lysis buffer, we further demonstrated that addition of GSH-dEE but not of GSH-mEE to BSO-treated CXR3 cells was able to restore the oxidation of hXRCC3 cysteine residues after UVA_MEMi_ radiation (Figure 6D). We obtained similar results using the human cell line MRC5Vi (Supplemental Figures S6A and S6B). We also found that the intracellular GSH protects the cells from the photocytotoxic effects of UVA_MEMi_ radiation (Figure 6E).

### hXRCC3 Oxidation does not Require Intracellular GSH in Response to Oxidizing Agents others than UVA_MEMi_

To test whether GSH-dependent hXRCC3 oxidation is restricted to photosensitization by UVA radiation or might be induced by other oxidants, untreated or BSO-treated CXR3 cells were exposed to increasing concentrations of tert-butyl hydroperoxide (tButH_2_O_2_), menadione (MN) that generates intracellular superoxide *via* redox cycling [48], rufloxacine followed by UVA radiation in PBS (UVA_RFX_) that generates mainly singlet oxygen and hydroxyl radical by photosensitization [49], 2,4-dinitrochlorobenzene (DNCB) that is an alkylating agent used to deplete GSH pool and an irreversible inhibitor of thioredoxine reductase [50], and diamide, a thiol-specific oxidant [51]. We then analysed the formation of prot-SSG adducts and the oxidation of hXRCC3 in response to these oxidants.

**Figure 7:**
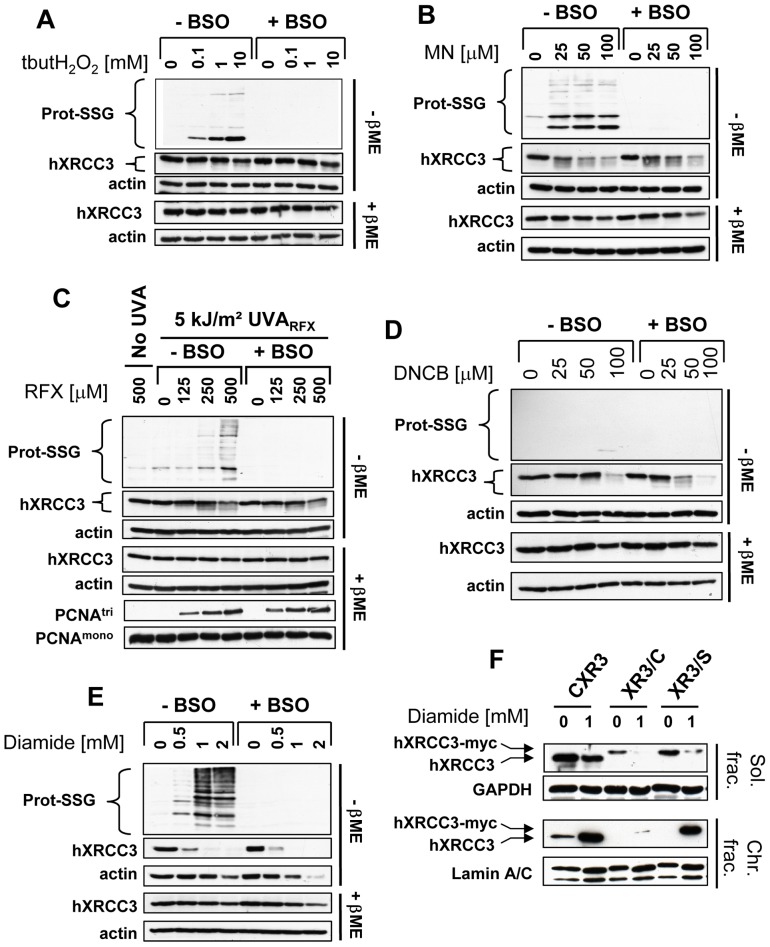
GSH/GSSG pool is not required for hXRCC3 oxidation in CHO cells in response to oxidants other than UVA_MEMi_. CXR3 cells treated or not with BSO were exposed to increasing concentrations of (**A**) tButH_2_O_2_ for 30 min, (**B**) menadione (MN) for 10 min, (**C**) UVA+Rufloxacine (UVA_RFX_), (**D**) 1,4-dinitrochlorobenzene (DNCB) for 30 min and (**E**) diamide for 30 min. Immediately post treatments, cells were lysed in buffer containing 10 mM NEM and protein extracts analysed by Western blot in non-reducing (-ßME) and reducing (+ßME) conditions. Prot-SSG adducts, hXRCC3, PCNA and actin were detected using anti-GSH, anti-XRCC3, anti-PCNA, and anti-actin antibodies, respectively. PCNA^mono^ = monomeric PCNA; PCNA^tri^ = covalently bound trimeric PCNA. ßME: ß-mercaptoethanol. (**F**) CXR3, XR3/C and XR3/S cells were exposed to 1 mM diamide for 30 min, and soluble (Sol. frac.) and chromatine (Chr. frac.) fractions were recovered. hXRCC3 and hXRCC3-myc tagged proteins were detected using anti-XRCC3 antibody. GAPDH and Lamin A/C were used as loading control.

As shown in Figure 7, S-glutathionylation was detected only in GSH proficient cells (-BSO) in response to all treatments (Figures 7A, 7B, 7C and 7E), with the exception of DNCB (Figure 7D) that probably alkylates GSH before it can make mixed disulfides with endogenous proteins. We observed the oxidation of hXRCC3 thiols in response to all treatments but the relative amount of oxidized hXRCC3 greatly depends on the oxidizing agent. For example, tButH_2_O_2_ (Figure 7A) appeared to be a weak oxidant with regards to hXRCC3 oxidation, even at the highest concentration of 10 mM, which contrasts with the strong oxidazing effect observed with 25 µM of MN (Figure 7B). Most importantly, formation of oxidized hXRCC3 induced by all the used agents was not prevented by lowering the level of GSH/GSSG pool (Figure 7, condition +BSO). In response to DNCB, hXRCC3 oxidation was even more pronounced in GSH-deficient cells if compared to GSH-proficient cells (Figure 7D). Unexpectedly and unlike what we observed in cells exposed to UVA_MEMi_, the photosensitization of RFX by UVA (UVA_RFX_) also led to a GSH-independent hXRCC3 oxidation despite ^1^O_2_ production as shown by the oxidation of PCNA in these cells (Figure 7C). Moreover, NaN_3_ and L-Histidine, but not NAC, prevented hXRCC3 oxidation induced by UVA_RFX_, thus supporting a key role of ^1^O_2_ in this process (Supplemental Figure S7).

We also noticed that in non reducing condition, the hXRCC3 protein is barely detected in cells treated with 100 µM DNCB and not detected in cells treated with diamide at concentrations ≥1 mM (Figures 7D and 7E, condition -ßME). In contrast, hXRCC3 is clearly detectable in reducing condition although a slight decrease in the total level of soluble hXRCC3 is observed at these concentrations if compared to untreated samples (Figures 7D and 7E, condition +ßME). Such differences in the immunodetection of hXRCC3 between reducing and non reducing conditions were not observed in diamide-treated cells expressing XR3/S mutated protein (Figure S8A), suggesting that the formation of intra and/or intermolecular disulfide bonds in hXRCC3 by diamide hides the epitope (amino acids 315 to 346) recognized by anti-XRCC3 antibody. Furthermore, we found that a fraction of hXRCC3 protein relocalizes to the chromatin in response to diamide by a mechanism that does not depend on its cysteine residues (Figure 7F), even though hXRCC3 activity is not required for cell survival in response to this chemical (Figure S8B).

### MN Induces hXRCC3 Oxidation and its Relocalization at the Chromatin

**Figure 8:**
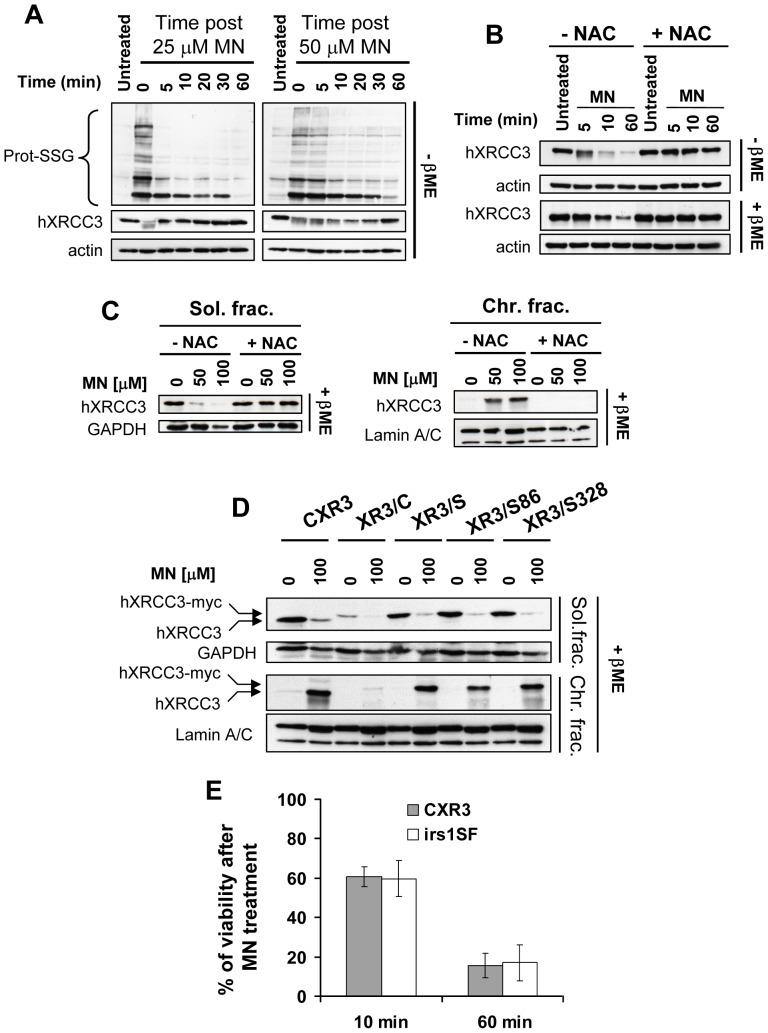
N-acetyl-L-cystein prevents hXRCC3 oxidation and its relocalization at the chromatin in response to MN. (**A**) CXR3 cells were exposed to 25 and 50 µM MN for 10 min at 37°C. S-glutathionylated proteins and oxidation of hXRCC3 protein were analyzed by Western blot at different time points after treatment. XRCC3, prot-SSG, and actin were detected using anti-XRCC3, anti-GSH, or anti-actin antibodies, respectively. (**B**) CXR3 cells were exposed to 50 µM MN in the presence or not of 10 mM NAC and hXRCC3 protein oxidation was analyzed at different time points after treatment by Western blot. (**C**) CXR3 cells were exposed to 50 and 100 µM MN for 1h in the presence or not of 10 mM NAC. (**D**) Cells expressing wild-type (CXR3, XR3/C) or mutated (XR3/S, XR3/S86 and XR3/S328) hXRCC3 were exposed to 100 µM MN for 1h. In (**C**) and (**D**), the soluble (Sol. frac.) and chromatine (Chr. frac.) fractions were recovered immediately post treatment. hXRCC3 and hXRCC3-myc tagged proteins were analysed by Western blot using anti-XRCC3 antibody. GAPDH and Lamin A/C were used as loading control. ßME: ß-mercaptoethanol. (**E**) XRCC3 proficient (CXR3) and deficient (irs1SF) cells were exposed to 100 µM MN for 10 and 60 min. Cell viability was assessed 24 h post treatment by MTT assay. Values are the mean +/− SD of 5 independent experiments.

As MN induces S-glutathionylation (Figure 7B, see also [52]) and hXRCC3 oxidation in CHO cells, we further investigated the reversion of both modifications by Western blot. At first, CXR3 cells were treated with 25 and 50 µM MN for 10 min and then incubated in drug-free complete medium for increasing periods of time. The reduction rate relied on the initial concentration of MN (Figure 8A). Indeed, oxidized hXRCC3 is reduced within 5 min after the release from 25 µM MN while it persisted up to 30 min after 50 µM MN (Figure 8A). Similarly, the rate of S-deglutathionylation was faster after the release from 25 than from 50 µM MN (Figure 8A). These data demonstrate that the formation of intra- and inter-molecular disulfide bridges triggered by MN are fully reversed in CHO cells after drug removal.

Next, CHO cells were treated with 50 µM MN for increasing periods of time in the presence or not of NAC to scavenge MN-induced ROS [53]. We found that hXRCC3 is oxidized after 5 min of treatment and that the level of soluble hXRCC3 protein is severely decreased after 60 min (Figure 8B, condition –NAC). This decrease corresponds to a relocalization of the protein at the chromatin fraction upon a prolonged period of MN treatment (Figure 8C, condition -NAC), relocalization that does not require the cysteine residues of hXRCC3 (Figure 8D). NAC efficiently prevented both oxidation of the protein and its relocalization (Figures 8B and 8C, condition +NAC). Although a slight decrease in the expression level of hXRCC3 was observed in MN-treated MRC5Vi cells, we did not observe relocalization of the protein at the chromatin (Figure S9 and data not shown). Finally, we analysed the viability of XRCC3 proficient (CXR3) and deficient (irs1SF) CHO cells exposed to 100 µM MN for 10 and 60 min, and found that XRCC3 does not contribute to the cell survival after MN (Figure 8E).

## Discussion

The cysteine residues at the surface of proteins are potentially targeted by ROS resulting in oxidative modifications of their thiol side chain (*e.g.* intra and/or inter disulfide bonds formation) that can be important to regulate positively or negatively the protein activity [54]. For example, activity of the DNA repair protein hOgg1 is regulated in response to oxidative stress by oxidation of critical cysteine residues [55], and the substitution from serine 326 to cysteine 326 (S326C) in hOgg1, a frequently occurring polymorphism in the human population, alters its DNA repair capacity due to oxidation of Cys326 [56]–[58]. While investigating a possible role of the DNA repair protein hXRCC3 in the inhibition of DNA synthesis triggered by UVA-induced ROS [34], we discovered that cysteine residues of hXRCC3 were oxidized. In this study, we bring the first evidences that the redox state of the protein depends on the intracellular environment and on the type of oxidizing agents.

The oxidation of sulfhydryl moieties of hXRCC3 by various oxidizing agents was investigated in human and hamster cells, the latter expressing hXRCC3 under the control of its own promoter [30]. Thus, any differences in the oxidation state of hXRCC3 between the two types of cell lines cannot be attributed to a difference in the amino acid sequence of the protein. In this study, oxidation of hXRCC3 was investigated using an XRCC3 antibody that recognizes an epitope surrounding Cys328. According to the model we computed, it appears that 6 out of the 8 cysteine residues of hXRCC3 (cysteines 86, 141, 193, 221, 310 and 328) are accessible to the solvent, and are, except Cys141, spatially close to well conserved arginine or lysine residues that could contribute to the ionization of the cysteine thiols, thus rendering them more reactive and susceptible to oxidation [22].

As a first surprising result, we found that the sensitivity of hXRCC3 to oxidation is less marked in human transformed or immortalized cells (MRC5Vi, HaCaT, and HCT116) than in CHO cells. Furthermore, in CHO cells exposed to oxidative stress, we observed changes in the electrophoretic mobility of the protein, while, under the same conditions, we observed a slight decrease in the immunodetection of the protein in human cells. This indicates i) that the sensitivity of cysteine thiols of hXRCC3 to oxidation is influenced by the intracellular environment, and ii) that the antibody is sensitive to the steric hindrance of Cys328. In favour of this latter hypothesis, we observed that the conjugation of malPEG, a bulky alkylating agent, to the cysteine thiols of hXRCC3 renders hXRCC3 undetectable by Western blot in both CHO and human cells. In contrast, the conjugation of NEM, a small alkylating agent, does not impact on hXRCC3 immunodetection. Furthermore, diamide that promotes the formation of disulfide bridges also led to loss of immunodetection of the protein in non reducing conditions but not in reducing conditions. We propose that in human cells the oxidative stress favours the formation of a disulfide bridge involving Cys328 and another cysteine, thus masking the epitope. One likely candidate is Cys86 insofar as our *in silico* model of hXRCC3 structure suggests that the closest C_α_-C_α_ distance is between Cys86 and Cys328. In CHO cells, the formation of a disulfide bridge involving Cys328 is not favoured, but others are formed as mutation of Cys328 to Ser328 still leads to changes in the electrophoretic mobility of the protein. The difference in Cys328 reactivity in human *versus* CHO cells may be attributed to a change in hXRCC3 conformation due to a different intracellular environment - *i.e.* ATP and Ca^2+^ concentration, the presence of molecular chaperones, the pH, the solvent, the intracellular macromolecular concentration, the balance of antioxidant/oxidant - all features influencing protein folding [59], [60]. Alternatively, the complex formed by hXRCC3 with its partner Rad51C [27], [61], [62] might be slightly different in the heterologous system (human XRCC3 interacting with hamster Rad51C) if compared to the homologous one (human XRCC3 interacting with human Rad51C). We cannot exclude that the sulfhydryl group of cysteines of hXRCC3 undergoes others forms of oxidation, especially irreversible oxidations (*i.e.* to sulfinic and sulfonic acid), and that others amino acids can also be oxidized [10], oxidations that are not detected by our experimental approach. This point is particularly relevant insofar as the use of malPEG revealed that all accessible thiols are potentially oxidized in both human and CHO cells. We also found that the kinetic of reduction of disulfide bonds in hXRCC3 depends on the intensity of the oxidative stress. In cells exposed to 160 kJ/m^2^ UVA_MEMi_ or 25 µM menadione for 10 min, disulfide bonds are *de novo* reduced in few minutes post treatment, while this time is sustained following exposure of cells to 50 µM menadione for 10 min.

In this study, we have also shown that wild-type (hXRCC3 and XR3/C) and mutated proteins (XR3/S, XR3/S86 and XR3/S328) are similarly distributed between the cytoplasm and the nucleus in untreated cells. It has been shown that XRCC3 stability is dependent on heterodimerization with Rad51C [63], [64], thus suggesting that XR3/S mutant might still proficient for Rad51C interaction. In response to CPT, we didn’t observe an increased accumulation of the proteins in the nucleus, nor did we detect hXRCC3 protein associated with the chromatin (data not shown). Our results are in agreement with previously published data showing that the subcellular distribution of hXRCC3 is not significantly affected by DNA damage, and that the amount of hXRCC3 at the chromatin can be very low after damage, depending on the cell type [64]. However, despite a protein localisation similar to that of wild-type, the XR3/S protein, but not XR3/S86 or XR3/S328 proteins, confers CPT sensitivity to the cells showing that one or more cysteine residues, other than Cys86 and Cys328, are critical for hXRCC3 activity. Recently, Somyajit et coll. have reported that hXRCC3 is phosphorylated on Ser225 by ATR in an ATM signaling pathway [65]. These authors showed that hXRCC3 phosphorylation is required for chromatin loading of Rad51 and HR-mediated repair of double-strand breaks [65]. As Ser225 is close to Cys221, it is possible that Cys221 contribute to the ATM- and ATR-mediated phosphorylation of hXRCC3.

We discovered that hXRCC3 expressed in CHO localizes almost exclusively at the chromatin in response to a severe oxidative stress (*e.g.* 1 mM diamide for 30min or 50 µM menadione for 1h), by a mechanism that does not require cysteine residues of hXRCC3. Further studies are required to understand i) the biological function of this relocalization in response to an oxidative stress in our particular cellular model (CXR3 cells), ii) why such relocalization was not observed in the human transformed fibroblasts MRC5Vi, and iii) whether or not this relocalization can affect HR-dependent DNA repair.

Another striking finding of our study was that different mechanisms are involved in the formation of disulfide bridges in hXRCC3. Indeed, GSH facilitates disulfide-bond formation in hXRCC3 in response to UVA_MEMi_ but not UVA_RFX_. This is quite unexpected since both conditions of irradiation trigger ^1^O_2_ production, which is essential for hXRCC3 oxidation. This discrepancy remains unclear and might reflect the importance of subcellular localization of the photosensitizer. Indeed, the lifetime (τ_Δ_) and diffusion length (dl) of ^1^O_2_ were estimated to be relatively short in cells; τ_Δ_ around 0.5 µs or less (0.05 µs) and dl around 20 nm [9], [66]. These values indicate that ^1^O_2_ reacts with biomolecules that are in its close vicinity. Previous reports have established the chemical quenching of ^1^O_2_ by the thiolate group of cysteines (-S^−^) resulting in the formation of disulfide bonds [67], [68]. Therefore, we hypothesize that in mammalian cells, ^1^O_2_ generated by UVA_RFX_ reacts immediately with the thiol groups of hXRCC3, while ^1^O_2_ generated by UVA_MEMi_ oxidizes GSH to GSSG, which in turn oxidizes hXRCC3. So far, GSH-dependent oxidation of hXRCC3 is unique to UVA_MEMi_ radiation since we did not observe it with other oxidizing agents, such as menadione (MN), 2,4-dinitrochlorobenzene (DNCB) or tert-butyl hydroperoxide (tButH_2_O_2_).

We have previously reported that UVA-induced ROS impinge on DNA replication through a mechanism that does not require functional DNA integrity checkpoint pathways [34]. Our data indicate that hXRCC3 is not involved in this mechanism, although HR is stimulated by UVA-induced ROS. At cell toxicity around 50–70% (80 kJ/m^2^ UVA_MEMi_, 10 J/m^2^ UVC and 2 Gy for γ-rays), UVA_MEMi_ is 3 times less efficient to stimulate recombination than UVC but 4 to 7 times more efficient than γ-rays (compare present results and [36], [69]). UVA_MEMi_-induced recombination may be explained by the presence of complex type of lesions that might obstruct replication fork progression, and that require HR repair to be further processed [31]. Interestingly, Somyajit et al. have found that XRCC3 is required for the HR-mediated recovery of collapsed forks but is dispensable for the restart of stalled forks [65]. Our observation that HR does not efficiently protect asynchronous cells from the lethal action of UVA radiation is in agreement with our previous findings in the yeasts *Saccharomyces cerevisiae* and *Schizosaccharomyces pombe* [33], [70]. However, we cannot exclude that HR is in part required for survival upon UVA exposure of cells in S-phase, as previously reported [33].

The work describing in this paper raises the question about the functionality of the oxidized hXRCC3 protein: what are the consequences if cells are subjected to an oxidative stress while exposed to DNA damaging agent that generates damages repaired by HR? Further studies are required to answer this question.

HR is a multi-step DNA repair pathway that involves more than 10 different proteins, including members of the Rad51 family [26]. The human proteins Rad51 (UniProt Q06609), XRCC2 (UniProt O43543), RAD51L1 (UniProt O15315), RAD51L2 (UniProt O43502) and RAD51L3 (UniProt O75771) contain between 5 and 12 cysteines. Our observation that cysteine residues of hXRCC3 are sensitive to ROS raises the possibility that cysteines of those others proteins involved in HR may also be sensitive to the cellular redox state.

## Materials and Methods

### Cell Lines and Silencing of Gene Expression (siRNA)

Chinese hamster irs1SF cell line is XRCC3-deficient and CXR3 cells are irs1SF cells stably complemented with human XRCC3 from a cosmid library [30]. The CHO-K1 DRA10 cell line contains an intrachromosomal recombination substrate composed of two inactive copies of the neomycin-resistant gene [35]. MRC5Vi (transformed fibroblasts) [34], HaCaT (immortalized keratinocytes) [71] and HCT116 (colorectal carcinoma cells) [72] are human cell lines. Eagle’s Minimum Essential Medium (MEM) with Earle’s salts containing phenol red and L-glutamine, MEM without phenol red and L-glutamine (MEMi), L-glutamine (L-glu) 100X, penicillin 10000 UI/Streptomycin 10000 µg 100X (P/S), non-essential amino acid (NEAA) 100X, and sodium pyruvate (100X) were from Eurobio (France). Fetal bovine serum (FBS) was from PAA (France). CHO, MRC5Vi, HaCaT and HCT116 were grown in 10% FBS Eagle’s MEM containing P/S 1X, L-glu 1X, NEAA 1X and sodium pyruvate 1X at 37°C, 95% humidity and 5% CO_2_. Transfection of human *XRCC3* siRNA (*siXRCC3*, ON-TARGETplus Set of 4, Dharmacon) and RNAi negative control low GC (*siCtr*, Invitrogen Life Technology) was performed in OptiMEM®I using INTERFERin™ (Polyplus-transfection, Ozyme, France). The final concentration of siRNA was 10 nM and experiments were carried out 48 h post transfection.

### Reagents

Camptothecin (CPT) was dissolved in DMSO. N-ethylmaleimide (NEM), menadione (MN), glutathione di-ethylester (GSH-dEE), 5-(and-6)-chloromethyl-2′7′-dichlorodihydrofluorescein diacetate acetyl ester (CM-H2DCFDA) and 2,4-dinitrochlorobenzene (DNCB) were dissolved in EtOH. L-Histidine, buthionine sulfoximine (BSO), N-acetyl-L-cysteine (NAC), glutathione mono-ethylester (GSH-mEE), diamide and sodium azide (NaN_3_) were dissolved in H_2_O. tert-Butyl hydroperoxide (tButH_2_O_2_) is at 70% in aqueous solution. Methoxypolyethylene glycol maleimide (malPEG) was directly dissolved in lysis buffer. 3-(4,5-Dimethylthiazol-2-yl)-2,5-diphenyltetrazolium bromide (MTT) and rufloxacine (RFX) were dissolved in phosphate buffered saline (PBS). All chemicals were purchased from Sigma-Aldrich (France), except for GSH-dEE that was purchased from Bachem (Switzerland) and CM-H2DCFDA from Invitrogen (France). RFX was supplied by Pr G. De Guidi (Dipartimento di Scienze Chimiche, Catania, Italy).

### Cloning of Human *XRCC3* into Mammalian Expression Vectors and Generation of XRCC3 Cys→Ser Mutants

Wild-type human *XRCC3* cDNA was amplified by PCR from pEF6/V5-His-XRCC3 [kindly provided by L. Thompson, see ref. [73]] and cloned into pCI(puro)myc [74], leading to pXR3/C. Cysteine to serine (Cys to Ser) mutations were introduced using the QuickChange® site-directed mutagenesis kit (Stratagene) and led to pXR3/S86 (Cys86 to Ser86), pXR3/S328 (Cys328 to Ser328) or pXR3/S (all Cys to Ser). Sequences were confirmed by direct sequencing (GATC Biotech).

### Stable Transfection into irs1SF Cells

Mammalian expression vectors pCI(puro)myc, pXR3/C, pXR3/S86, pXR3/S328 and pXR3/S were transfected into irs1SF cells using jetPEI™ transfection reagent (Polyplus-transfection SA, France). Forty-eight hours post transfection, cells were incubated for 12 to 14 days in the presence of 10 µg/ml puromycine (Invivogen, Cayla SAS, France). For each stable transfection, few clones were analysed for myc-tagged hXRCC3 expression.

### Treatments with Pro- and Anti-oxidants and Recovery of Soluble and Insoluble Protein Extracts

Excepted for experiments with RFX, in all other experiments cells were exposed to UVA radiation in phenol red-free MEMi medium (UVA_MEMi_) at a fluency rate of 50 mW/cm^2^, as previously described [34]. RFX was added to MEMi medium 1 h prior to UVA radiation in drug-free PBS 1×(UVA_RFX_; fluency rate  = 10 mW/cm^2^). MN was added directly to the culture medium while incubation with DNCB, tButH_2_O_2_ and diamide was performed in MEMi. All incubations were at 37°C. NAC and NaN_3_ were added during the treatment. GSH-mEE and GSH-dEE were added for 60 min and BSO for 24 h before exposure of cells to the oxidants. Following treatments, cells in dishes were washed with cold PBS and lysed on ice for 5 min in lysis buffer [10 mM Hepes, pH 7.5, 100 mM NaCl, 300 mM sucrose, 3 mM MgCl_2_, 1 mM EGTA, 50 mM NaF, 20 mM ß-glycerophosphate, 0.3% triton X-100, 0.1 mM sodium orthovanadate and complete mini EDTA-free protease inhibitors (Roche Diagnosis)]. When indicated, 10 mM NEM or 4 mM malPEG were added to the lysis buffer to react with thiol groups. The supernatant correspond to the total soluble protein extracts that were precipitated with acetone. After removal of acetone and air drying, the pellets were dissolved in 1.5 X SDS loading buffer (150 mM Tris-HCl, pH 6.8, 3% SDS, 0.15% bromophenol blue and 15% glycerol) without ß-mercaptoethanol (−ß). Protein extracts were denatured for 5 min at 95°C and 150 mM ß-mercaptoethanol was added to half of the sample (+ß). Insoluble material (chromatin-bound fraction) was recovered by scraping off the nuclear matrix in PBS. After centrifugation at 4°C, insoluble fractions were resuspended in 1.5 X SDS loading buffer containing ß-mercaptoethanol, denatured for 15 min at 95°C and centrifuged for 30 min at room temperature to pellet any solids.

### Cellular Fractionati on

To fractionate the cells into cytoplasm and nucleus, CPT-treated cells were washed with cold PBS and incubated for 5 min on ice in hypotonic buffer [10 mM Hepes, pH 7.5, 1.5 mM MgCl_2_, 10 mM KCl, 0.5 mM DTT and complete mini EDTA-free protease inhibitors (Roche Diagnosis)]. The cells were scraped off from the culture dishes and open cells were broken using a pre-chilled Dounce homogenizer to release nuclei. After centrifugation, the supernatant is retained as the cytoplasmic fraction, and the pellet as the nuclear fraction. The nuclear pellet is washed one time in sucrose buffer (250 mM sucrose, 10 mM MgCl_2_ and complete mini EDTA-free protease inhibitors), resuspended in 1.5 X SDS loading buffer containing ß-mercaptoethanol, denatured for 15 min at 95°C and centrifuged for 30 min at room temperature to pellet any solids.

### Western Blot Analysis

Twenty to thirty micrograms of protein extract were separated on 9% SDS-PAGE and transferred onto PROTRAN^®^ nitrocellulose membrane (Whatman) using a Trans-Blot Semi-Dry apparatus (Bio-Rad Laboratories). Membranes were probed with the following primary antibodies: rabbit polyclonal anti-XRCC3 antibody (Novus Biologicals clone 100–165), rabbit polyclonal anti-myc (A14), mouse anti-PCNA (PC10), mouse anti-Lamin A/C (E-1), and mouse anti-GAPDH (A-3) antibodies (Santa Cruz Biotechnology), mouse monoclonal anti-actin (clone AC-15) (Sigma-Aldrich), mouse monoclonal anti-GSH (clone D8) (GeneTex). The membranes were then probed with the appropriate peroxidase-conjugated secondary antibody and developed using either the ECL™ Western blotting Detection Reagents (Amersham Biosciences) or the WesternBright™ ECL-spray (ECL^+^, Advansta, France). Primary and secondary antibodie were prepared in Tris buffered saline (TBS) containing 5% bovine serum albumin (BSA) and 0.05% tween 20. The autoradiographies were scanned on a Pro48 scanner (PFU, Japan) controlled by the *SilverFast Ai* scan software (LaserSoft Imaging AG, Germany). All quantifications were further done using Image J.

### Quantification of Intracellular Level of GSH

Cells plated at 1.5×10^4^ per well in a 96-well plate were incubated overnight and BSO was added for further 24 hours. When indicated, GSH-mEE or GSH-dEE was added for 60 min to BSO-treated cells. After washing the cells 3 times with PBS 1X, quantification of intracellular levels of GSH was performed using GSH-Glo™ Glutathione Assay (Promega). Luminescence data were recorded on a Wallac 1420 Multilabel Counter (Perkin Elmer).

### Measurement of Intracellular ROS

The incubation of cells with the cell-permeant indicator for ROS, 5-(and-6)-chloromethyl-2′,7′-dichlorodihydrofluorescein diacetate acetyl ester (CM-H2DCFDA, Invitrogen France), exposure to UVA radiation and analysis of fluorescent by flow cytometry were performed as previously described [34].

### Cell Viability

To assess for cell viability in response to CPT, 4×10^5^ cells per well were seeded in 6-well dishes, incubated overnight at 37°C and exposed to 10 nM CPT for 16 h. Cell viability was measured 48 h post CPT treatment. To assess for cell viability in response to UVA or MN, 5 to 10×10^4^ MRC5Vi cells were plated in 40 mm dishes, incubated overnight at 37°C and then exposed to UVA or MN as described above. Cell viability was assessed 24 hours post treatment. To perform the MTT assay, culture medium was replaced by fresh culture medium containing 0.5 mg/ml thiazolblue Tetrazoliumbromide and cells were incubated at 37°C until purple precipitate is visible. The resulting intracellular purple formazan was then solubilized in the dark for 2 h in isopropanol 95%/0.4 N HCl. Spectrophotometric quantification was performed at 570 nm.

### Clonogenic Assay

CHO cells in exponentially growing phase were irradiated at various doses of UVA in phenol red-free MEMi (UVA_MEMi_) or PBS (UVA_PBS_). Following irradiation, cells were incubated at 37°C for 24 h in fresh complete medium, trypsinized, counted and replated at low density. The cultures were kept in the incubator for 8–10 days and colonies were vizualized by incubating the cells in 2% methylene blue (MB) for 3–5 min. The colonies were rinced with water, air dried, and counted (>50 cells per colonie).

### Homologous Recombination Assay

CHO DRA10 cells were irradiated as described above, incubated at 37°C in fresh complete medium for 24 h, trypsinized, and divided into two fractions. The first fraction was used to calculate the viability by measuring the plating efficiency. The second fraction was plated at high density in selective medium containing 500 µg/ml of G418 to select for G418 resistant cells. Colonies were stained with MB and counted.

### BrdU Incorporation and Cell Cycle Analysis

The BrdU incorporation and cell cycle analysis were performed as previously reported [34]. Briefly, pulse-labeled BrdU cells were exposed to UVA radiation, allowed to recover for 6 h at 37°C before being fixed in ethanol, treated with FITC-conjugated anti-BrdU antibody to label S-phase cells and with propidium iodide (PI) to stain for DNA content. All samples were analysed by a FACScalibur flow cytometer (Becton Dickinson).

### Computational Modeling of hXRCC3

The three-dimensional structure of human XRCC3 has been computed with Modeller software [75]. The crystal structures of the archaeal RadA from *Sulfolobus solfataricus* (PDB code 2DFL) and from *Methanococcus voltae* (PDB code 1XU4), two Rad51 homolog template structures chosen to generate this model were solved at less than 2.9 Å resolution [76], [77] and displayed more than 30 percent sequence identity with human XRCC3. RAD51 from *Saccharomyces cerevisiae* (PDB code 3LDA), the third structure model used in the computations, display 30 percent sequence identity with human XRCC3 and was solved at less than 2.0 Å resolution [78]. The multiple sequence alignment of the archaeal RadA from *Sulfolobus solfataricus*, the archaeal RadA from *Methanococcus voltae*, the RAD51 protein of *Saccharomyces cerevisiae* and the human XRCC3 was performed with ClustalW [79] software and refined manually. A structure-based alignment of all the sequences of the template models was done prior to the final alignment.

## Supporting Information

Figure S1 **The inhibition of DNA synthesis induced by UVA photosensitization is not abolished in XRCC3 deficient human cells.** Western blot analysis of XRCC3 (**A** and **B**) and S-phase delay (**C** and **D**) in XRCC3 proficient and deficient cells. MRC5Vi cells were transfected with control (siCtr) or XRCC3 (siXRCC3) siRNA **(A** and **C)**, while XRCC3 gene was disrupted in HCT116 cells **(B** and **D)**. (**A** and **B**) Total soluble protein extracts were prepared in lysis buffer containing 10 mM NEM. hXRCC3 was detected using a polyclonal anti-XRCC3 antibody (Novus Biologicals). The star (*) indicates non specific cross-reactivity of the antibody. (**C** and **D**) Cells were pulse-labeled with BrdU for 30 min, exposed to UVA_MEMi_ and further incubated at 37°C for 6 h. S-phase cells were dectected using FITC-conjugated anti-BrdU antibody and the DNA was stained by propidium iodide (PI). Samples were analysed by flow cytometry.
(TIF)

Figure S2 **Reduced level of hXRCC3 immunodetection after UVA radiation in the three human cell lines.** (A) MRC5Vi, HaCaT and HCT116 cells were exposed to various doses of UVA_MEMi_ and total soluble protein extracts were prepared immediately post radiation in lysis buffer containing 10 mM NEM. hXRCC3 was detected using a polyclonal anti-XRCC3 antibody (Novus Biologicals). (**B**) MRC5Vi cells were treated with 160 kJ/m^2^ UVA_MEMi_ and protein extracts were prepared at various time points post radiation. The blot is representative of 3 independent experiments. (**C**) The relative level of reduced hXRCC3 in condition (B) was calculated by dividing the intensity of hXRCC3 band in UVA-treated cells by the intensity of the same band in unirradiated cells. The star (*) indicates non specific cross-reactivity of the antibody. Statistical analysis was performed using ANOVA with TUKEY’s post test. *P<0.05.
(TIF)

Figure S3 **NaN_3_ but not NAC prevents oxidation of hXRCC3 by UVA photosensitization in MRC5Vi.** Human MRC5Vi cells were exposed to 160 kJ/m^2^ UVA in MEMi in the presence of increasing concentration of NaN_3_ (**A**) or NAC (**B**). Cells were lysed immediately post radiation and hXRCC3 was detected using a polyclonal anti-XRCC3 antibody (Novus Biologicals). ßME: ß-mercaptoethanol.
(TIF)

Figure S4 **UVA photosensitization induces ROS in BSO-treated cells.** CXR3 cells were pre-incubated or not with 0.5 mM BSO for 24 h. (**A**) Cell viability was then assessed by MTT assay. Results are the mean ± SD of 3 independent experiments. (**B**) Cells treated or not with BSO were incubated with 10 µM of the ROS probe CM-H2DCFDA for 30 min prior to irradiation at 160 kJ/m^2^ UVA in probe-free MEMi. Following irradiation, the cells were incubated at 37°C for 30 min in the presence of the ROS probe, and the fluorescence was analyzed by FACS. (**C**) Untreated and BSO-treated cells were exposed to 160 kJ/m^2^ UVA_MEMi_ and lysed immediately post radiation. Samples were analyzed by Western blot in reducing conditions (+ßME). PCNA antibody detects monomeric (PCNA^mono^) and covalently bound trimeric (PCNA^tri^). ßME: ß-mercaptoethanol.
(TIF)

Figure S5 **The conjugation of malPEG to hXRCC3, GAPDH and PCNA prevents their immunodetection by Western blot.** XRCC3 proficient (CXR3) and deficient (irs1SF) cells were lysed in lysis buffer containing 10 mM NEM or 4 mM malPEG. Thirty micrograms of total soluble protein extracts were analysed by Western blot in reducing conditions. (**A**) Ponceau red staining of the membrane. XRCC3 (**B**) GAPDH (**C**) or PCNA (**D**) proteins were detected using anti-XRCC3, anti-GAPDH, and anti-PCNA antibodies, respectively. Note that hXRCC3-malPEG, GAPDH-malPEG and PCNA-malPEG conjugates are not or barely detected by XRCC3, GAPDH and PCNA antibodies, respectively.
(TIF)

Figure S6 **GSH-dEE restores hXRCC3 oxidation in response to UVA radiation in BSO-treated MRC5Vi cells.** MRC5Vi cells were pre-incubated for 24 h in culture medium containing or not 0.5 mM BSO. Thereafter, BSO-treated cells were complemented with 2 mM GSH-dEE or GSH-mEE for 1 h. (**A**) Measurement of GSH level in cells. Values are expressed as % of GSH relative to control cells (–BSO) and results are the mean ± SD of 3 independent experiments. (**B**) Cells treated as described in panel A were exposed to 160 kJ/m^2^ UVA_MEMi_ and lysed immediately post radiation in buffer containing 4 mM malPEG. hXRCC3 was analysed by Western blot in reducing conditions (+ß-mercaptoethanol).
(TIF)

Figure S7 **Oxidation of hXRCC3 by UVA radiation in the presence of Rufloxacin is prevented by NaN_3_ or L-Histidine but not NAC in CHO cells.** CXR3 cells were incubated for 1 h in MEMi with 500 µM Rufloxacin (RFX). Thereafter, cells were irradiated at 160 kJ/m^2^ UVA (fluency rate  = 10 mW/cm^2^) in RFX-free PBS containing or not 10 mM sodium azide (NaN_3_), 50 mM L-Histidine (L-His) or 10 mM N-acetyl-L-cysteine (NAC). Total soluble protein extracts were prepared immediately post UVA and samples were analysed by Western blot in non reducing (− ßME) or reducing (+ ßME) conditions. Actin was used as loading control.
(TIF)

Figure S8 **hXRCC3 does not protect the cells against diamide toxicity.** (A) Cells expressing XR3/S protein were pre-incubated for 24 h in culture medium containing or not 0.5 mM BSO. Thereafter, the cells were exposed to increasing concentration of diamide, and total soluble protein extracts prepared immediately post treatment. The expression level of S-glutathionylated and of XR3/S proteins was analysed by Western blot in non reducing (− ßME) or reducing (+ ßME) conditions. Actin was used as loading control. (**B**) CXR3 cells and irs1SF cells complemented with wild type hXRCC3-myc (XR3/C), with hXRCC3-myc mutated at all cysteines (XR3/S) or with empty vector (EV) was exposed to 1 mM diamide for 30 min. The cell viability was assessed 24 h post treatment by MTT assay. Values are the mean +/− SD of 3 independent experiments.
(TIF)

Figure S9 **MN induces hXRCC3 oxidation in MRC5Vi cells.** MRC5Vi cells were either incubated for 10 min (**A**) or for the indicated periods of time (**B**) with 100 µM MN in the presence or not of 10 mM NAC. Thereafter, the cells were lysed in buffer containing 10 mM NEM and the samples analysed by Western blot in non reducing (− ßME) or reducing (+ ßME) conditions. The star (*) indicates non-specific cross reactivity of the antibody.
(TIF)

## References

[1] Ridley AJ, Whiteside JR, McMillan TJ, Allinson SL (2009) Cellular and sub-cellular responses to UVA in relation to carcinogenesis. Int J Radiat Biol 85: 177–195. 19296341 10.1080/09553000902740150

[2] Pattison DI, Rahmanto AS, Davies MJ (2012) Photo-oxidation of proteins. Photochem Photobiol Sci 11: 38–53. 21858349 10.1039/c1pp05164d

[3] Sage E, Girard PM, Francesconi S (2012) Unravelling UVA-induced mutagenesis. Photochem Photobiol Sci 11: 74–80. 21901217 10.1039/c1pp05219e

[4] Baier J, Maisch T, Maier M, Engel E, Landthaler M, et al (2006) Singlet oxygen generation by UVA light exposure of endogenous photosensitizers. Biophys J 91: 1452–1459. 16751234 10.1529/biophysj.106.082388 PMC1518628

[5] Bracchitta G, Catalfo A, Martineau S, Sage E, De Guidi G, et al (2013) Investigation of the phototoxicity and cytotoxicity of naproxen, a non-steroidal anti-inflammatory drug, in human fibroblasts. Photochem Photobiol Sci 12: 911–922. 23478633 10.1039/c3pp25326k

[6] Pryor WA, Houk KN, Foote CS, Fukuto JM, Ignarro LJ, et al (2006) Free radical biology and medicine: it's a gas, man! Am J Physiol Regul Integr Comp Physiol. 291: R491–511. 10.1152/ajpregu.00614.2005 16627692

[7] Girotti AW, Kriska T (2004) Role of lipid hydroperoxides in photo-oxidative stress signaling. Antioxid Redox Signal 6: 301–310. 15025931 10.1089/152308604322899369

[8] Redmond RW, Kochevar IE (2006) Spatially resolved cellular responses to singlet oxygen. Photochem Photobiol Sci 82: 1178–1186. 10.1562/2006-04-14-IR-874 16740059

[9] Schweitzer C, Schmidt R (2003) Physical mechanisms of generation and deactivation of singlet oxygen. Chem Rev 103: 1685–1757. 12744692 10.1021/cr010371d

[10] Davies MJ (2004) Reactive species formed on proteins exposed to singlet oxygen. Photochem Photobiol Sci 3: 17–25. 14743273 10.1039/b307576c

[11] Maresca V, Flori E, Briganti S, Camera E, Cario-Andre M, et al (2006) UVA-induced modification of catalase charge properties in the epidermis is correlated with the skin phototype. J Invest Dermatol 126: 182–190. 16417235 10.1038/sj.jid.5700021

[12] Montaner B, O'Donovan P, Reelfs O, Perrett CM, Zhang X, et al (2007) Reactive oxygen-mediated damage to a human DNA replication and repair protein. EMBO Rep 8: 1074–1079. 17932513 10.1038/sj.embor.7401084 PMC2247395

[13] von Montfort C, Sharov VS, Metzger S, Schoneich C, Sies H, et al (2006) Singlet oxygen inactivates protein tyrosine phosphatase-1B by oxidation of the active site cysteine. Biol Chem 387: 1399–1404. 17081112 10.1515/BC.2006.175

[14] Reddie KG, Carroll KS (2008) Expanding the functional diversity of proteins through cysteine oxidation. Curr Opin Chem Biol 12: 746–754. 18804173 10.1016/j.cbpa.2008.07.028

[15] Wan L, Ottinger E, Cho S, Dreyfuss G (2008) Inactivation of the SMN complex by oxidative stress. Mol Cell 31: 244–254. 18657506 10.1016/j.molcel.2008.06.004 PMC2867055

[16] Guo Z, Kozlov S, Lavin MF, Person MD, Paull TT (2010) ATM activation by oxidative stress. Science 330: 517–521. 20966255 10.1126/science.1192912

[17] Meyer Y, Buchanan BB, Vignols F, Reichheld JP (2009) Thioredoxins and glutaredoxins: unifying elements in redox biology. Annu Rev Genet 43: 335–367. 19691428 10.1146/annurev-genet-102108-134201

[18] Lillig CH, Holmgren A (2007) Thioredoxin and related molecules–from biology to health and disease. Antioxid Redox Signal 9: 25–47. 17115886 10.1089/ars.2007.9.25

[19] Shelton MD, Chock PB, Mieyal JJ (2005) Glutaredoxin: role in reversible protein s-glutathionylation and regulation of redox signal transduction and protein translocation. Antioxid Redox Signal 7: 348–366. 15706083 10.1089/ars.2005.7.348

[20] Gallogly MM, Starke DW, Mieyal JJ (2009) Mechanistic and Kinetic Details of Thiol-Disulfide Exchange by Glutaredoxins and Potential Mechanisms of Regulation. Antioxid Redox Signal 11: 1059–1081. 19119916 10.1089/ars.2008.2291 PMC2842129

[21] Meister A (1995) Glutathione metabolism. Methods Enzymol 251: 3–7. 7651209 10.1016/0076-6879(95)51106-7

[22] Dalle-Donne I, Milzani A, Gagliano N, Colombo R, Giustarini D, et al (2008) Molecular mechanisms and potential clinical significance of S-glutathionylation. Antioxid Redox Signal 10: 445–473. 18092936 10.1089/ars.2007.1716

[23] Ghezzi P, Bonetto V, Fratelli M (2005) Thiol-disulfide balance: from the concept of oxidative stress to that of redox regulation. Antioxid Redox Signal 7: 964–972. 15998251 10.1089/ars.2005.7.964

[24] Shackelford RE, Heinloth AN, Heard SC, Paules RS (2005) Cellular and molecular targets of protein S-glutathiolation. Antioxid Redox Signal 7: 940–950. 15998249 10.1089/ars.2005.7.940

[25] Dalle-Donne I, Rossi R, Colombo G, Giustarini D, Milzani A (2009) Protein S-glutathionylation: a regulatory device from bacteria to humans. Trends Biochem Sci 34: 85–96. 19135374 10.1016/j.tibs.2008.11.002

[26] Thacker J (2005) The RAD51 gene family, genetic instability and cancer. Cancer Lett 219: 125–135. 15723711 10.1016/j.canlet.2004.08.018

[27] Kurumizaka H, Ikawa S, Nakada M, Eda K, Kagawa W, et al (2001) Homologous-pairing activity of the human DNA-repair proteins Xrcc3.Rad51C. Proc Natl Acad Sci U S A 98: 5538–5543. 11331762 10.1073/pnas.091603098 PMC33248

[28] Masson JY, Tarsounas MC, Stasiak AZ, Stasiak A, Shah R, et al (2001) Identification and purification of two distinct complexes containing the five RAD51 paralogs. Genes Dev 15: 3296–3307. 11751635 10.1101/gad.947001 PMC312846

[29] Bishop DK, Ear U, Bhattacharyya A, Calderone C, Beckett M, et al (1998) Xrcc3 is required for assembly of Rad51 complexes in vivo. J Biol Chem 273: 21482–21488. 9705276 10.1074/jbc.273.34.21482

[30] Tebbs RS, Zhao Y, Tucker JD, Scheerer JB, Siciliano MJ, et al (1995) Correction of chromosomal instability and sensitivity to diverse mutagens by a cloned cDNA of the XRCC3 DNA repair gene. Proc Natl Acad Sci U S A 92: 6354–6358. 7603995 10.1073/pnas.92.14.6354 PMC41516

[31] Biverstal A, Johansson F, Jenssen D, Erixon K (2008) Cyclobutane pyrimidine dimers do not fully explain the mutagenicity induced by UVA in Chinese hamster cells. Mutat Res 648: 32–39. 18950648 10.1016/j.mrfmmm.2008.09.011

[32] Henry-Mowatt J, Jackson D, Masson JY, Johnson PA, Clements PM, et al (2003) XRCC3 and Rad51 modulate replication fork progression on damaged vertebrate chromosomes. Mol Cell 11: 1109–1117. 12718895 10.1016/s1097-2765(03)00132-1

[33] Dardalhon D, Angelin AR, Baldacci G, Sage E, Francesconi S (2008) Unconventional effects of UVA radiation on cell cycle progression in S. pombe. Cell Cycle 7: 611–622. 18256544 10.4161/cc.7.5.5400

[34] Girard PM, Pozzebon M, Delacote F, Douki T, Smirnova V, et al (2008) Inhibition of S-phase progression triggered by UVA-induced ROS does not require a functional DNA damage checkpoint response in mammalian cells. DNA Repair (Amst) 7: 1500–1516. 18603484 10.1016/j.dnarep.2008.05.004

[35] Liang F, Romanienko PJ, Weaver DT, Jeggo PA, Jasin M (1996) Chromosomal double-strand break repair in Ku80-deficient cells. Proc Natl Acad Sci U S A 93: 8929–8933. 8799130 10.1073/pnas.93.17.8929 PMC38571

[36] Lambert S, Lopez BS (2002) Inactivation of the RAD51 recombination pathway stimulates UV-induced mutagenesis in mammalian cells. Oncogene 21: 4065–4069. 12037689 10.1038/sj.onc.1205535

[37] Czaplewski C, Oldziej S, Liwo A, Scheraga HA (2004) Prediction of the structures of proteins with the UNRES force field, including dynamic formation and breaking of disulfide bonds. Protein Eng Des Sel 17: 29–36. 14985535 10.1093/protein/gzh003

[38] Hinz JM, Helleday T, Meuth M (2003) Reduced apoptotic response to camptothecin in CHO cells deficient in XRCC3. Carcinogenesis 24: 249–253. 12584174 10.1093/carcin/24.2.249

[39] Sonoda E, Zhao GY, Kohzaki M, Dhar PK, Kikuchi K, et al (2007) Collaborative roles of gammaH2AX and the Rad51 paralog Xrcc3 in homologous recombinational repair. DNA Repair (Amst) 6: 280–292. 17123873 10.1016/j.dnarep.2006.10.025

[40] Tyrrell RM, Pidoux M (1989) Singlet oxygen involvement in the inactivation of cultured human fibroblasts by UVA (334 nm, 365 nm) and near-visible (405 nm) radiations. Photochem Photobiol Sci 49: 407–412. 10.1111/j.1751-1097.1989.tb09187.x 2498913

[41] Bae SI, Zhao R, Snapka RM (2008) PCNA damage caused by antineoplastic drugs. Biochem Pharmacol 76: 1653–1668. 18823950 10.1016/j.bcp.2008.09.003 PMC2659951

[42] Aruoma OI, Halliwell B, Hoey BM, Butler J (1989) The antioxidant action of N-acetylcysteine: its reaction with hydrogen peroxide, hydroxyl radical, superoxide, and hypochlorous acid. Free Radic Biol Med 6: 593–597. 2546864 10.1016/0891-5849(89)90066-x

[43] Zafarullah M, Li WQ, Sylvester J, Ahmad M (2003) Molecular mechanisms of N-acetylcysteine actions. Cell Mol Life Sci 60: 6–20. 12613655 10.1007/s000180300001 PMC11138873

[44] Griffith OW, Meister A (1979) Potent and specific inhibition of glutathione synthesis by buthionine sulfoximine (S-n-butyl homocysteine sulfoximine). J Biol Chem 254: 7558–7560. 38242

[45] Noda T, Iwakiri R, Fujimoto K, Aw TY (2001) Induction of mild intracellular redox imbalance inhibits proliferation of CaCo-2 cells. FASEB J 15: 2131–2139. 11641239 10.1096/fj.01-0131com

[46] Levy EJ, Anderson ME, Meister A (1993) Transport of glutathione diethyl ester into human cells. Proc Natl Acad Sci U S A 90: 9171–9175. 8415673 10.1073/pnas.90.19.9171 PMC47524

[47] Minhas HS, Thornalley PJ (1995) Comparison of the delivery of reduced glutathione into P388D1 cells by reduced glutathione and its mono- and diethyl ester derivatives. Biochem Pharmacol 49: 1475–1482. 7763291 10.1016/0006-2952(94)00518-q

[48] Watanabe N, Dickinson DA, Liu RM, Forman HJ (2004) Quinones and glutathione metabolism. Methods Enzymol 378: 319–340. 15038978 10.1016/S0076-6879(04)78024-6

[49] Catalfo A, Scifo C, Stella S, Belvedere A, Renis M, et al (2005) Rufloxacin induced photosensitization in bio-models of increasing complexity. Photochem Photobiol Sci 4: 304–314. 15739000 10.1039/b406908k

[50] Arner ES, Bjornstedt M, Holmgren A (1995) 1-Chloro-2,4-dinitrobenzene is an irreversible inhibitor of human thioredoxin reductase. Loss of thioredoxin disulfide reductase activity is accompanied by a large increase in NADPH oxidase activity. J Biol Chem 270: 3479–3482. 7876079 10.1074/jbc.270.8.3479

[51] Kosower NS, Kosower EM (1995) Diamide: an oxidant probe for thiols. Methods Enzymol 251: 123–133. 7651192 10.1016/0076-6879(95)51116-4

[52] Bellomo G, Thor H, Orrenius S (1990) Modulation of cellular glutathione and protein thiol status during quinone metabolism. Methods Enzymol 186: 627–635. 2233322 10.1016/0076-6879(90)86158-r

[53] Criddle DN, Gillies S, Baumgartner-Wilson HK, Jaffar M, Chinje EC, et al (2006) Menadione-induced reactive oxygen species generation via redox cycling promotes apoptosis of murine pancreatic acinar cells. J Biol Chem 281: 40485–40492. 17088248 10.1074/jbc.M607704200

[54] Biswas S, Chida AS, Rahman I (2006) Redox modifications of protein-thiols: emerging roles in cell signaling. Biochem Pharmacol 71: 551–564. 16337153 10.1016/j.bcp.2005.10.044

[55] Bravard A, Vacher M, Gouget B, Coutant A, de Boisferon FH, et al (2006) Redox regulation of human OGG1 activity in response to cellular oxidative stress. Mol Cell Biol 26: 7430–7436. 16923968 10.1128/MCB.00624-06 PMC1636869

[56] Hill JW, Evans MK (2006) Dimerization and opposite base-dependent catalytic impairment of polymorphic S326C OGG1 glycosylase. Nucleic Acids Res 34: 1620–1632. 16549874 10.1093/nar/gkl060 PMC1405821

[57] Lee AJ, Hodges NJ, Chipman JK (2005) Interindividual variability in response to sodium dichromate-induced oxidative DNA damage: role of the Ser326Cys polymorphism in the DNA-repair protein of 8-oxo-7,8-dihydro-2′-deoxyguanosine DNA glycosylase 1. Cancer Epidemiol Biomarkers Prev 14: 497–505. 15734978 10.1158/1055-9965.EPI-04-0295

[58] Bravard A, Vacher M, Moritz E, Vaslin L, Hall J, et al (2009) Oxidation status of human OGG1-S326C polymorphic variant determines cellular DNA repair capacity. Cancer Res 69: 3642–3649. 19351836 10.1158/0008-5472.CAN-08-3943

[59] Stefani M (2008) Protein folding and misfolding on surfaces. Int J Mol Sci 9: 2515–2542. 19330090 10.3390/ijms9122515 PMC2635651

[60] Braakman I, Bulleid NJ (2011) Protein folding and modification in the mammalian endoplasmic reticulum. Annu Rev Biochem 80: 71–99. 21495850 10.1146/annurev-biochem-062209-093836

[61] Liu N, Schild D, Thelen MP, Thompson LH (2002) Involvement of Rad51C in two distinct protein complexes of Rad51 paralogs in human cells. Nucleic Acids Res 30: 1009–1015. 11842113 10.1093/nar/30.4.1009 PMC100342

[62] Masson JY, Stasiak AZ, Stasiak A, Benson FE, West SC (2001) Complex formation by the human RAD51C and XRCC3 recombination repair proteins. Proc Natl Acad Sci U S A 98: 8440–8446. 11459987 10.1073/pnas.111005698 PMC37455

[63] Lio YC, Schild D, Brenneman MA, Redpath JL, Chen DJ (2004) Human Rad51C deficiency destabilizes XRCC3, impairs recombination, and radiosensitizes S/G2-phase cells. J Biol Chem 279: 42313–42320. 15292210 10.1074/jbc.M405212200

[64] Gildemeister OS, Sage JM, Knight KL (2009) Cellular redistribution of Rad51 in response to DNA damage: novel role for Rad51C. J Biol Chem 284: 31945–31952. 19783859 10.1074/jbc.M109.024646 PMC2797266

[65] Somyajit K, Basavaraju S, Scully R, Nagaraju G (2013) ATM- and ATR-mediated phosphorylation of XRCC3 regulates DNA double-strand break-induced checkpoint activation and repair. Mol Cell Biol 33: 1830–1844. 23438602 10.1128/MCB.01521-12 PMC3624173

[66] Kanofsky JR (2011) Measurement of singlet-oxygen in vivo: progress and pitfalls. Photochem Photobiol 87: 14–17. 21143605 10.1111/j.1751-1097.2010.00855.x

[67] Buettner GR, Hall RD (1987) Superoxide, hydrogen peroxide and singlet oxygen in hematoporphyrin derivative-cysteine, -NADH and -light systems. Biochim Biophys Acta 923: 501–507. 3030441 10.1016/0304-4165(87)90060-2

[68] Devasagayam TP, Sundquist AR, Di Mascio P, Kaiser S, Sies H (1991) Activity of thiols as singlet molecular oxygen quenchers. J Photochem Photobiol B 9: 105–116. 1907641 10.1016/1011-1344(91)80008-6

[69] Lambert S, Lopez BS (2000) Characterization of mammalian RAD51 double strand break repair using non-lethal dominant-negative forms. EMBO J 19: 3090–3099. 10856252 10.1093/emboj/19.12.3090 PMC203369

[70] Kozmin S, Slezak G, Reynaud-Angelin A, Elie C, de Rycke Y, et al (2005) UVA radiation is highly mutagenic in cells that are unable to repair 7,8-dihydro-8-oxoguanine in Saccharomyces cerevisiae. Proc Natl Acad Sci U S A 102: 13538–13543. 16157879 10.1073/pnas.0504497102 PMC1224634

[71] Soeur J, Marrot L, Perez P, Iraqui I, Kienda G, et al (2011) Selective cytotoxicity of Aniba rosaeodora essential oil towards epidermoid cancer cells through induction of apoptosis. Mutat Res 718: 24–32. 21070863 10.1016/j.mrgentox.2010.10.009

[72] Yoshihara T, Ishida M, Kinomura A, Katsura M, Tsuruga T, et al (2004) XRCC3 deficiency results in a defect in recombination and increased endoreduplication in human cells. EMBO J 23: 670–680. 14749735 10.1038/sj.emboj.7600087 PMC1271813

[73] Yamada NA, Hinz JM, Kopf VL, Segalle KD, Thompson LH (2004) XRCC3 ATPase activity is required for normal XRCC3-Rad51C complex dynamics and homologous recombination. J Biol Chem 279: 23250–23254. 15037616 10.1074/jbc.M402247200

[74] Girard PM, Kysela B, Harer CJ, Doherty AJ, Jeggo PA (2004) Analysis of DNA ligase IV mutations found in LIG4 syndrome patients: the impact of two linked polymorphisms. Hum Mol Genet 13: 2369–2376. 15333585 10.1093/hmg/ddh274

[75] Marti-Renom MA, Stuart AC, Fiser A, Sanchez R, Melo F, et al (2000) Comparative protein structure modeling of genes and genomes. Annu Rev Biophys Biomol Struct 29: 291–325. 10940251 10.1146/annurev.biophys.29.1.291

[76] Wu Y, Qian X, He Y, Moya IA, Luo Y (2005) Crystal structure of an ATPase-active form of Rad51 homolog from Methanococcus voltae. Insights into potassium dependence. J Biol Chem 280: 722–728. 15537659 10.1074/jbc.M411093200

[77] Chen LT, Ko TP, Chang YC, Lin KA, Chang CS, et al (2007) Crystal structure of the left-handed archaeal RadA helical filament: identification of a functional motif for controlling quaternary structures and enzymatic functions of RecA family proteins. Nucleic Acids Res 35: 1787–1801. 17329376 10.1093/nar/gkl1131 PMC1874592

[78] Chen J, Villanueva N, Rould MA, Morrical SW (2010) Insights into the mechanism of Rad51 recombinase from the structure and properties of a filament interface mutant. Nucleic Acids Res 38: 4889–4906. 20371520 10.1093/nar/gkq209 PMC2919713

[79] Thompson JD, Higgins DG, Gibson TJ (1994) CLUSTAL W: improving the sensitivity of progressive multiple sequence alignment through sequence weighting, position-specific gap penalties and weight matrix choice. Nucleic Acids Res 22: 4673–4680. 7984417 10.1093/nar/22.22.4673 PMC308517

