# Supplementary figures and images for: Oxidative Stress in Mammalian Cells Impinges on the Cysteines Redox State of Human XRCC3 Protein and on Its Cellular Localization
Source: PLoS One. 2013 Oct 8;8(10):e75751. doi: 10.1371/journal.pone.0075751 (PMC3793007; doi:10.1371/journal.pone.0075751)

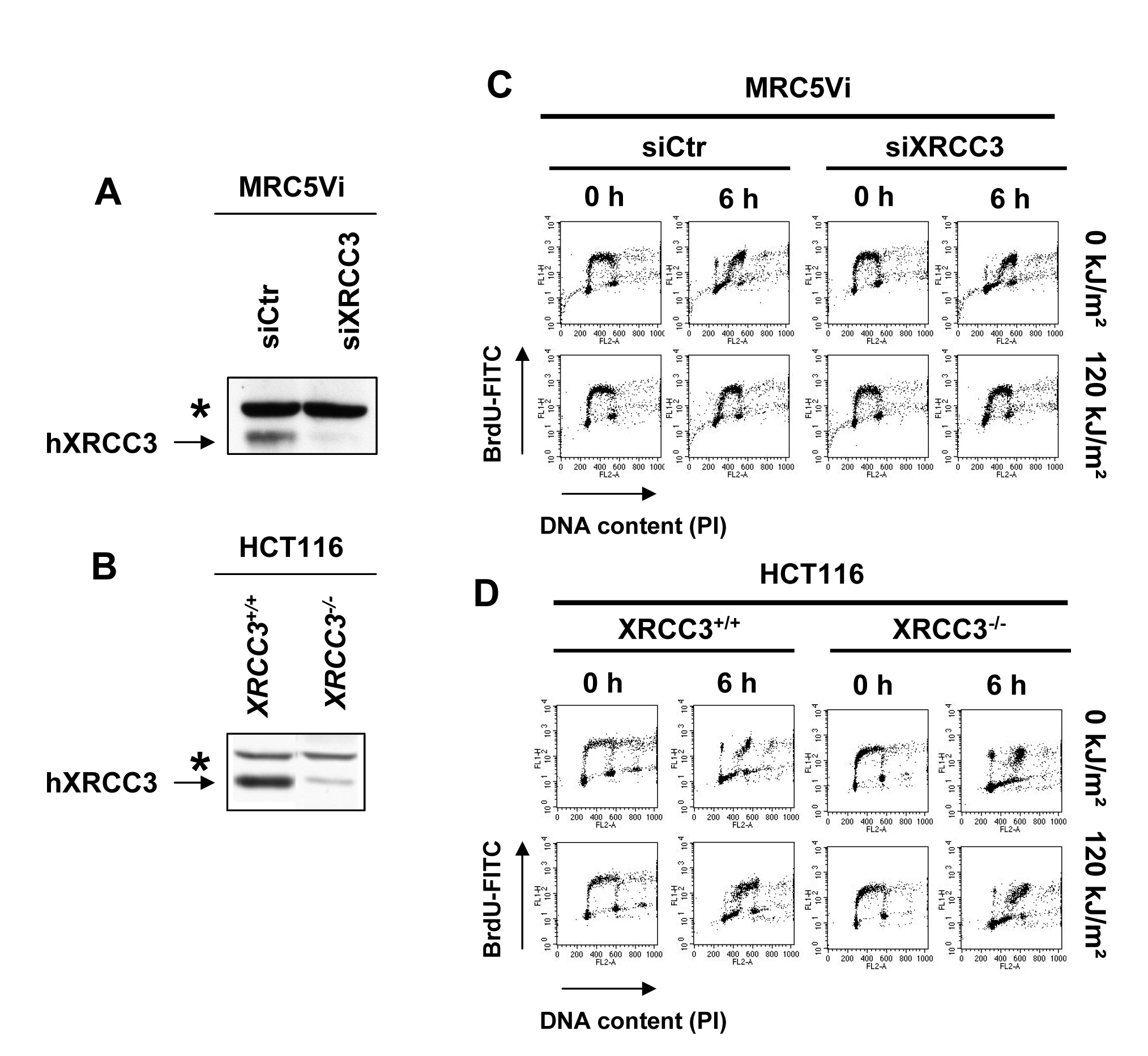

Supplement: Figure S1 — The inhibition of DNA synthesis induced by UVA photosensitization is not abolished in XRCC3 deficient human cells. Western blot analysis of XRCC3 (A and B) and S-phase delay (C and D) in XRCC3 proficient and deficient cells. MRC5Vi cells were transfected with control (siCtr) or XRCC3 (siXRCC3) siRNA (A and C), while XRCC3 gene was disrupted in HCT116 cells (B and D). (A and B) Total soluble protein extracts were prepared in lysis buffer containing 10 mM NEM. hXRCC3 was detected using a polyclonal anti-XRCC3 antibody (Novus Biologicals). The star (*) indicates non specific cross-reactivity of the antibody. (C and D) Cells were pulse-labeled with BrdU for 30 min, exposed to UVAMEMi and further incubated at 37°C for 6 h. S-phase cells were dectected using FITC-conjugated anti-BrdU antibody and the DNA was stained by propidium iodide (PI). Samples were analysed by flow cytometry. (TIF) [file pone.0075751.s001.tif]

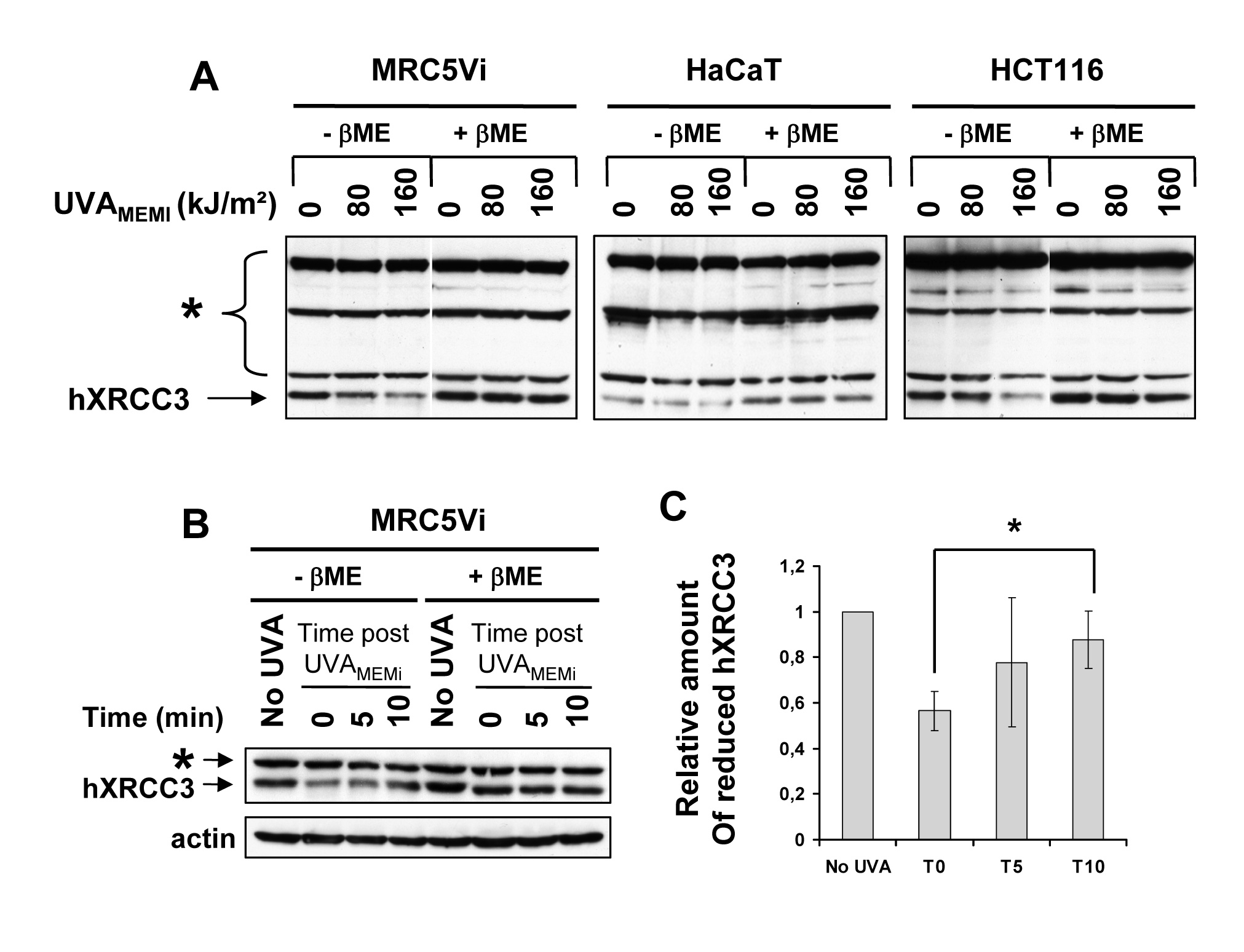

Supplement: Figure S2 — Reduced level of hXRCC3 immunodetection after UVA radiation in the three human cell lines. (A) MRC5Vi, HaCaT and HCT116 cells were exposed to various doses of UVAMEMi and total soluble protein extracts were prepared immediately post radiation in lysis buffer containing 10 mM NEM. hXRCC3 was detected using a polyclonal anti-XRCC3 antibody (Novus Biologicals). (B) MRC5Vi cells were treated with 160 kJ/m2 UVAMEMi and protein extracts were prepared at various time points post radiation. The blot is representative of 3 independent experiments. (C) The relative level of reduced hXRCC3 in condition (B) was calculated by dividing the intensity of hXRCC3 band in UVA-treated cells by the intensity of the same band in unirradiated cells. The star (*) indicates non specific cross-reactivity of the antibody. Statistical analysis was performed using ANOVA with TUKEY’s post test. *P<0.05. (TIF) [file pone.0075751.s002.tif]

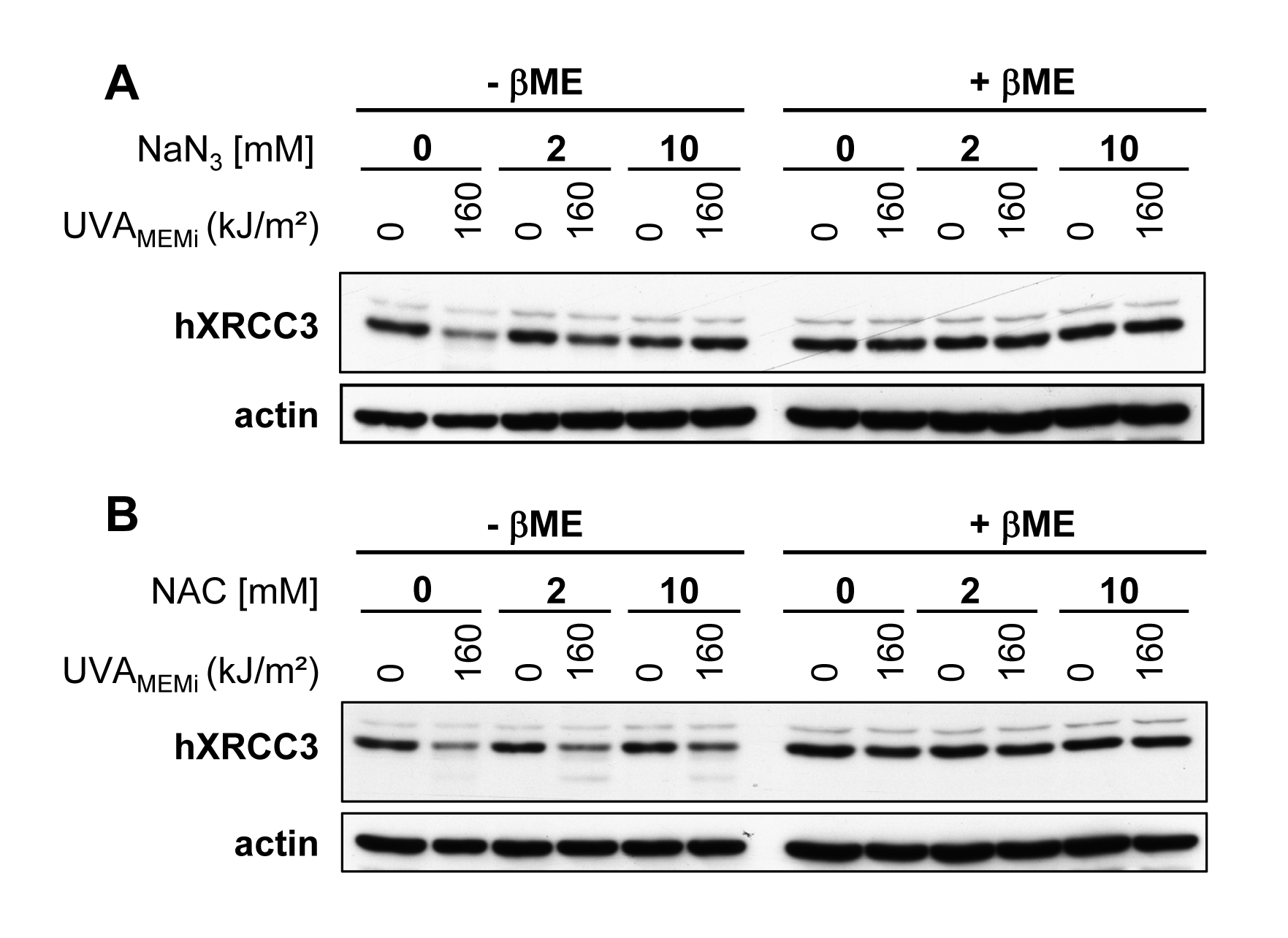

Supplement: Figure S3 — NaN3 but not NAC prevents oxidation of hXRCC3 by UVA photosensitization in MRC5Vi. Human MRC5Vi cells were exposed to 160 kJ/m2 UVA in MEMi in the presence of increasing concentration of NaN3 (A) or NAC (B). Cells were lysed immediately post radiation and hXRCC3 was detected using a polyclonal anti-XRCC3 antibody (Novus Biologicals). ßME: ß-mercaptoethanol. (TIF) [file pone.0075751.s003.tif]

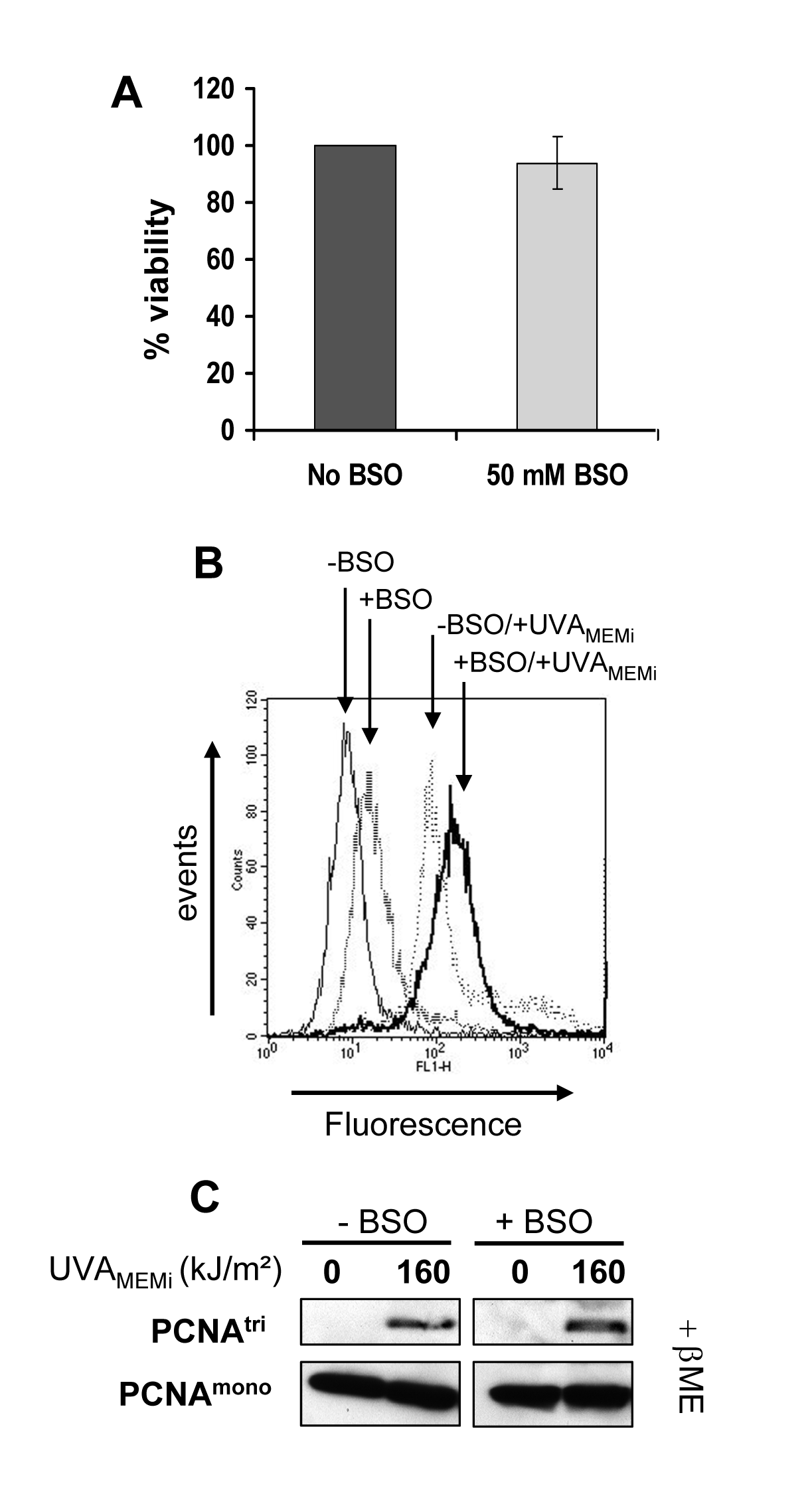

Supplement: Figure S4 — UVA photosensitization induces ROS in BSO-treated cells. CXR3 cells were pre-incubated or not with 0.5 mM BSO for 24 h. (A) Cell viability was then assessed by MTT assay. Results are the mean ± SD of 3 independent experiments. (B) Cells treated or not with BSO were incubated with 10 µM of the ROS probe CM-H2DCFDA for 30 min prior to irradiation at 160 kJ/m2 UVA in probe-free MEMi. Following irradiation, the cells were incubated at 37°C for 30 min in the presence of the ROS probe, and the fluorescence was analyzed by FACS. (C) Untreated and BSO-treated cells were exposed to 160 kJ/m2 UVAMEMi and lysed immediately post radiation. Samples were analyzed by Western blot in reducing conditions (+ßME). PCNA antibody detects monomeric (PCNAmono) and covalently bound trimeric (PCNAtri). ßME: ß-mercaptoethanol. (TIF) [file pone.0075751.s004.tif]

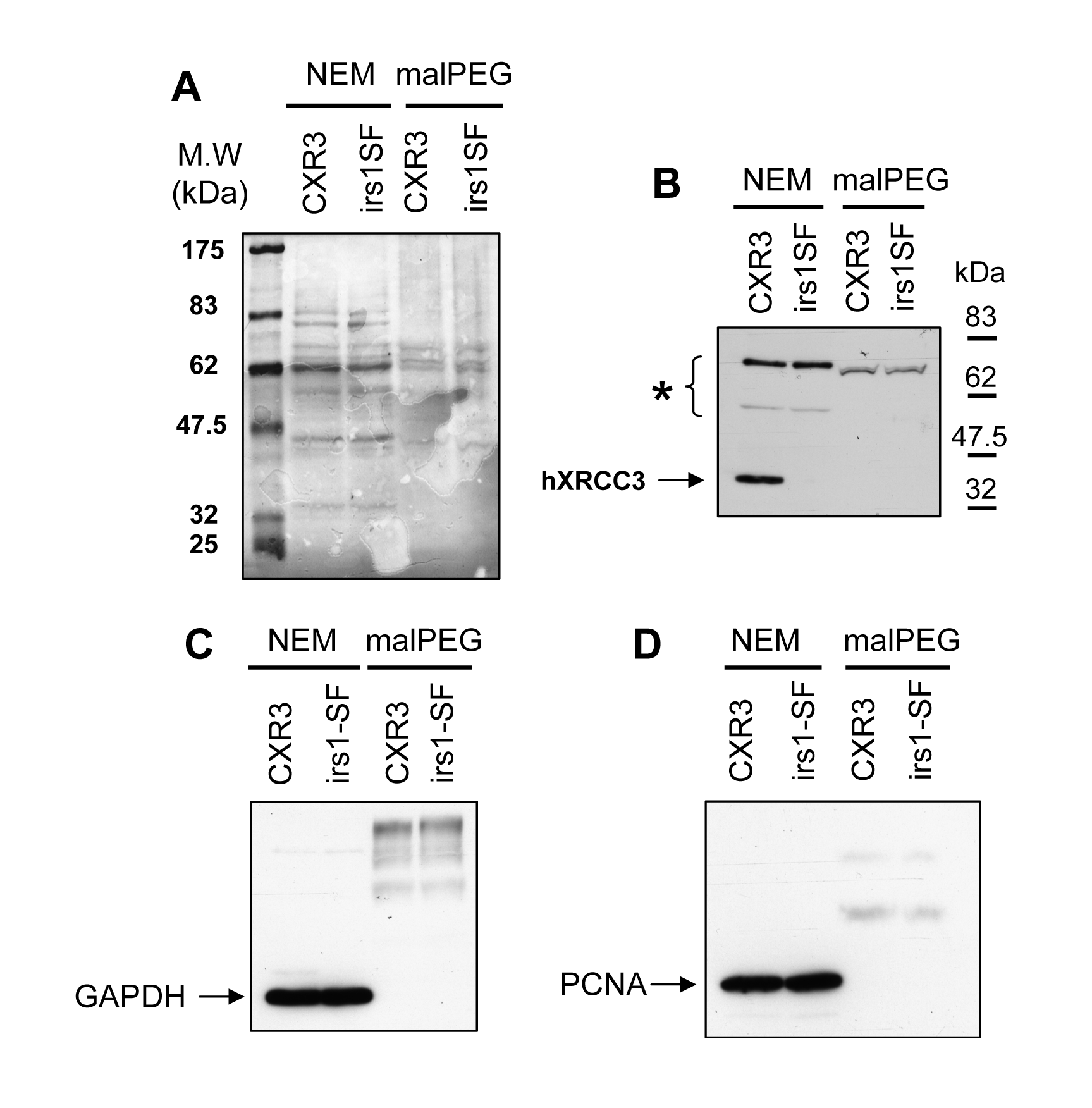

Supplement: Figure S5 — The conjugation of malPEG to hXRCC3, GAPDH and PCNA prevents their immunodetection by Western blot. XRCC3 proficient (CXR3) and deficient (irs1SF) cells were lysed in lysis buffer containing 10 mM NEM or 4 mM malPEG. Thirty micrograms of total soluble protein extracts were analysed by Western blot in reducing conditions. (A) Ponceau red staining of the membrane. XRCC3 (B) GAPDH (C) or PCNA (D) proteins were detected using anti-XRCC3, anti-GAPDH, and anti-PCNA antibodies, respectively. Note that hXRCC3-malPEG, GAPDH-malPEG and PCNA-malPEG conjugates are not or barely detected by XRCC3, GAPDH and PCNA antibodies, respectively. (TIF) [file pone.0075751.s005.tif]

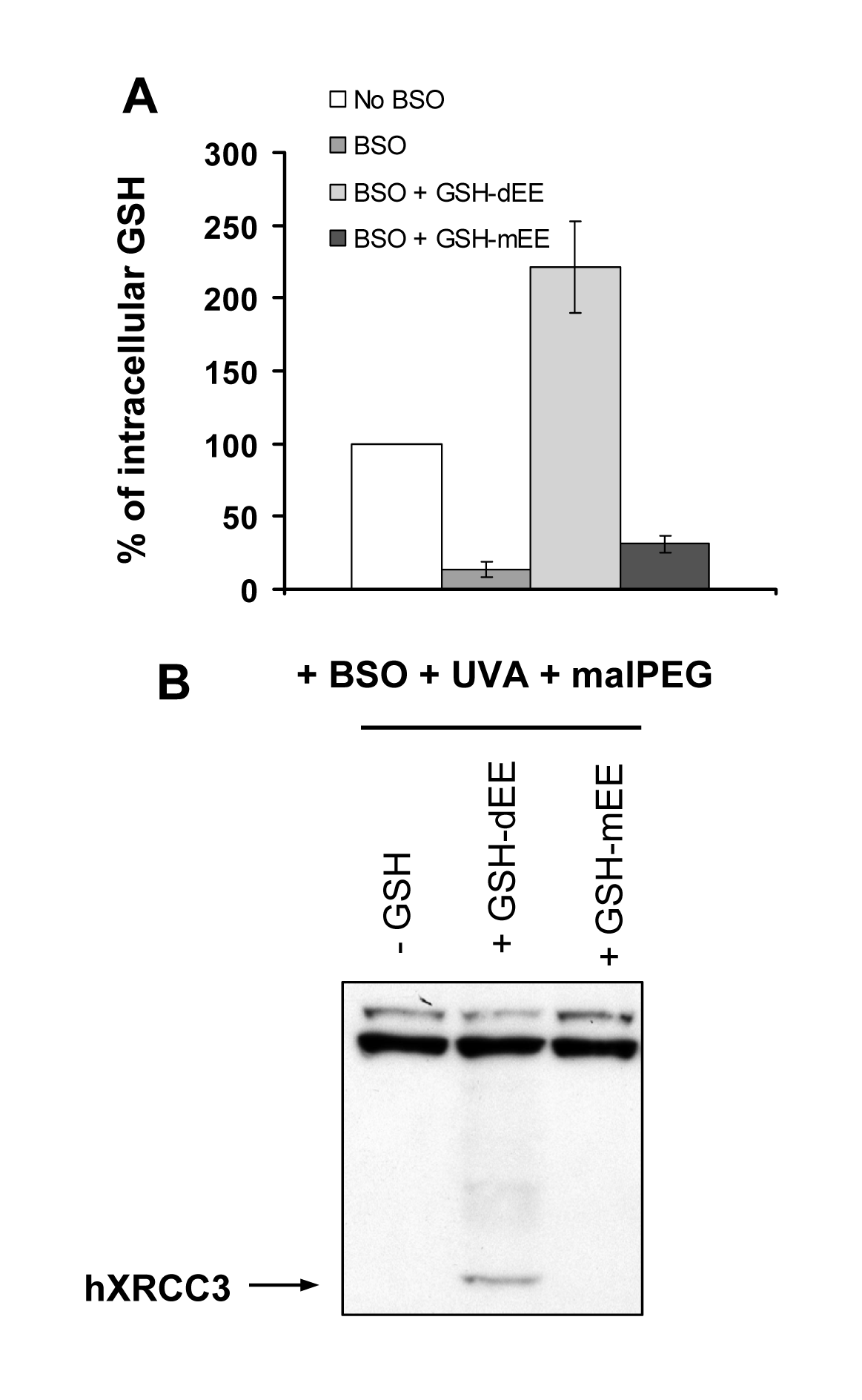

Supplement: Figure S6 — GSH-dEE restores hXRCC3 oxidation in response to UVA radiation in BSO-treated MRC5Vi cells. MRC5Vi cells were pre-incubated for 24 h in culture medium containing or not 0.5 mM BSO. Thereafter, BSO-treated cells were complemented with 2 mM GSH-dEE or GSH-mEE for 1 h. (A) Measurement of GSH level in cells. Values are expressed as % of GSH relative to control cells (–BSO) and results are the mean ± SD of 3 independent experiments. (B) Cells treated as described in panel A were exposed to 160 kJ/m2 UVAMEMi and lysed immediately post radiation in buffer containing 4 mM malPEG. hXRCC3 was analysed by Western blot in reducing conditions (+ß-mercaptoethanol). (TIF) [file pone.0075751.s006.tif]

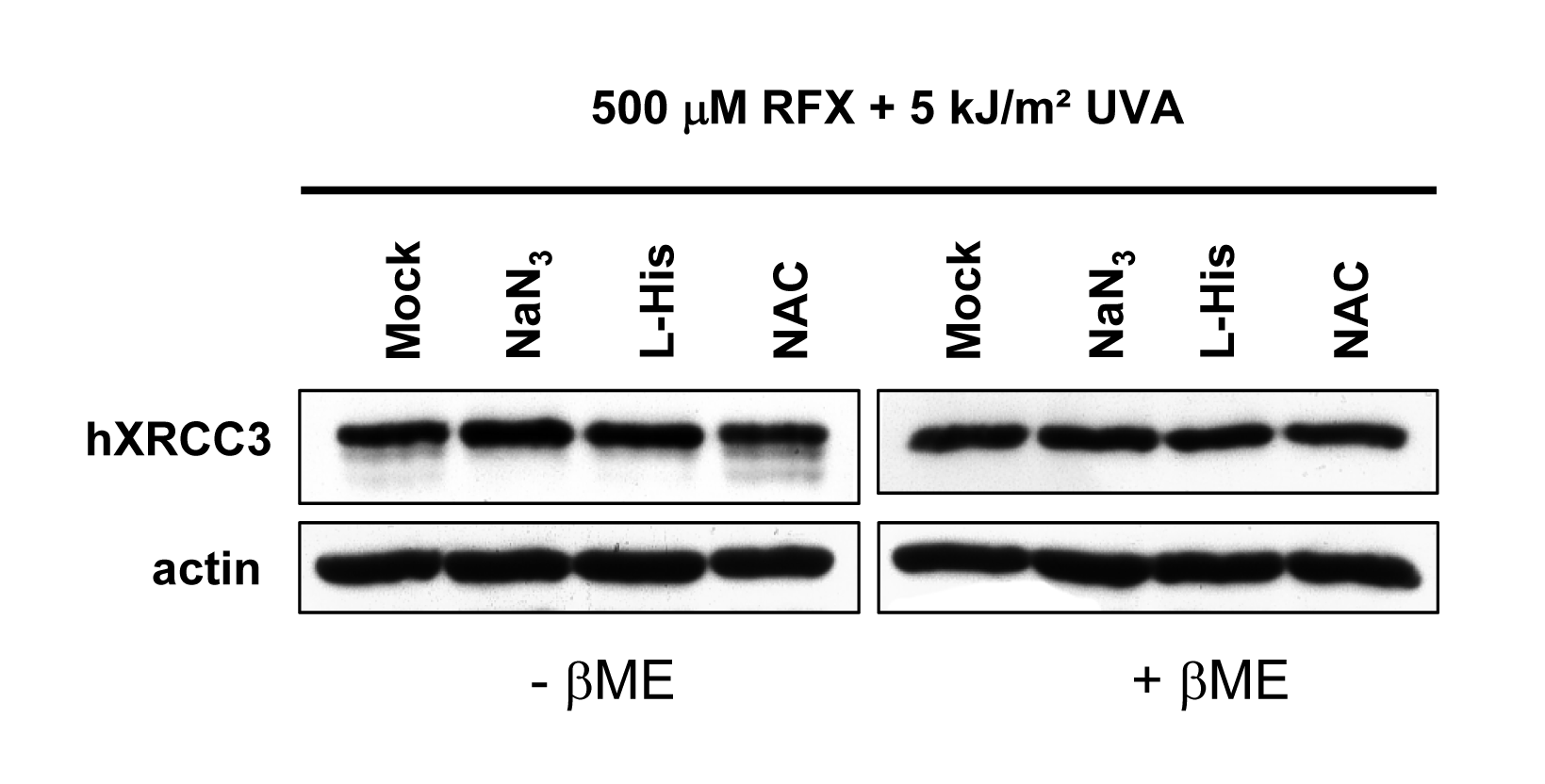

Supplement: Figure S7 — Oxidation of hXRCC3 by UVA radiation in the presence of Rufloxacin is prevented by NaN3 or L-Histidine but not NAC in CHO cells. CXR3 cells were incubated for 1 h in MEMi with 500 µM Rufloxacin (RFX). Thereafter, cells were irradiated at 160 kJ/m2 UVA (fluency rate = 10 mW/cm2) in RFX-free PBS containing or not 10 mM sodium azide (NaN3), 50 mM L-Histidine (L-His) or 10 mM N-acetyl-L-cysteine (NAC). Total soluble protein extracts were prepared immediately post UVA and samples were analysed by Western blot in non reducing (− ßME) or reducing (+ ßME) conditions. Actin was used as loading control. (TIF) [file pone.0075751.s007.tif]

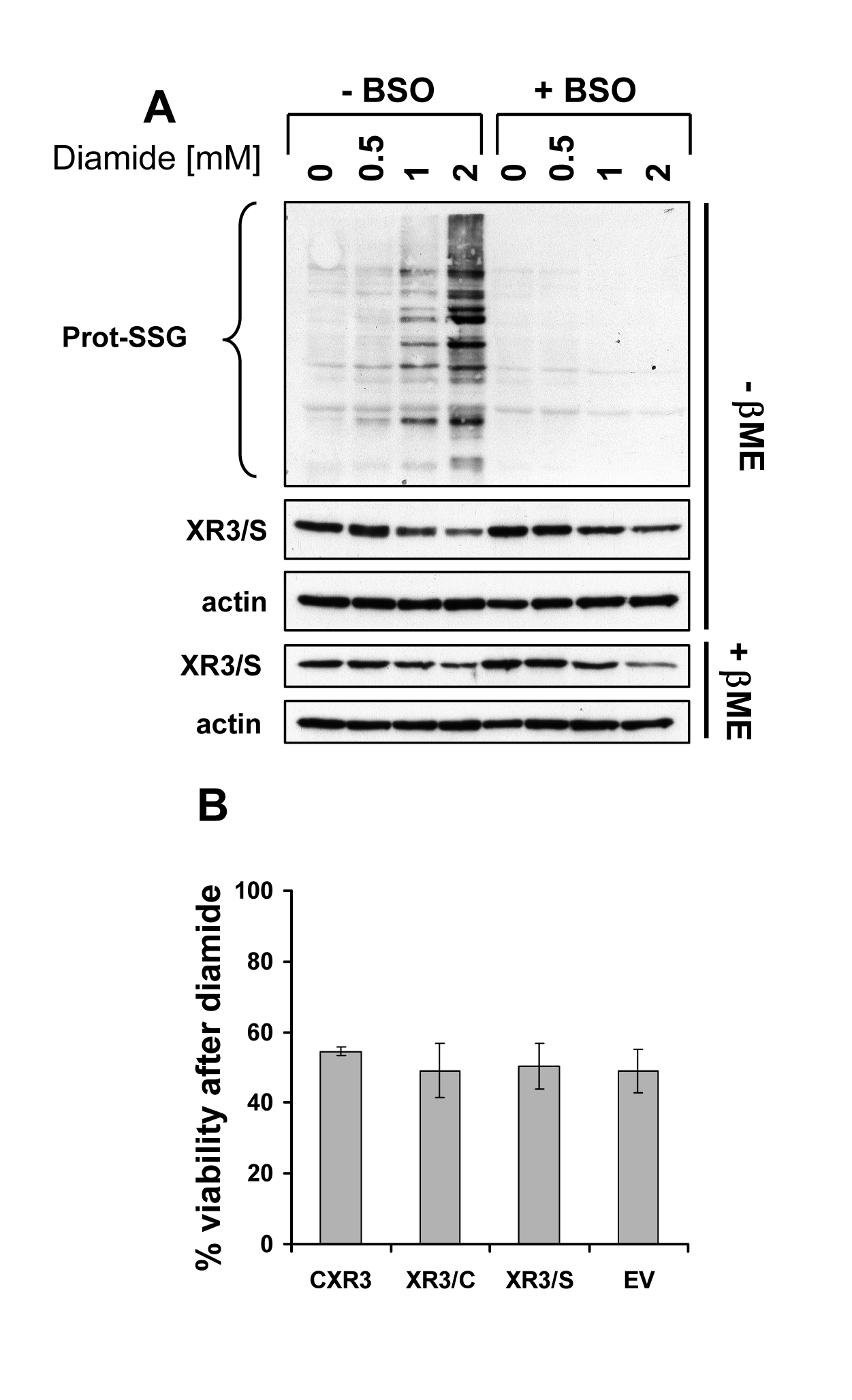

Supplement: Figure S8 — hXRCC3 does not protect the cells against diamide toxicity. (A) Cells expressing XR3/S protein were pre-incubated for 24 h in culture medium containing or not 0.5 mM BSO. Thereafter, the cells were exposed to increasing concentration of diamide, and total soluble protein extracts prepared immediately post treatment. The expression level of S-glutathionylated and of XR3/S proteins was analysed by Western blot in non reducing (− ßME) or reducing (+ ßME) conditions. Actin was used as loading control. (B) CXR3 cells and irs1SF cells complemented with wild type hXRCC3-myc (XR3/C), with hXRCC3-myc mutated at all cysteines (XR3/S) or with empty vector (EV) was exposed to 1 mM diamide for 30 min. The cell viability was assessed 24 h post treatment by MTT assay. Values are the mean +/− SD of 3 independent experiments. (TIF) [file pone.0075751.s008.tif]

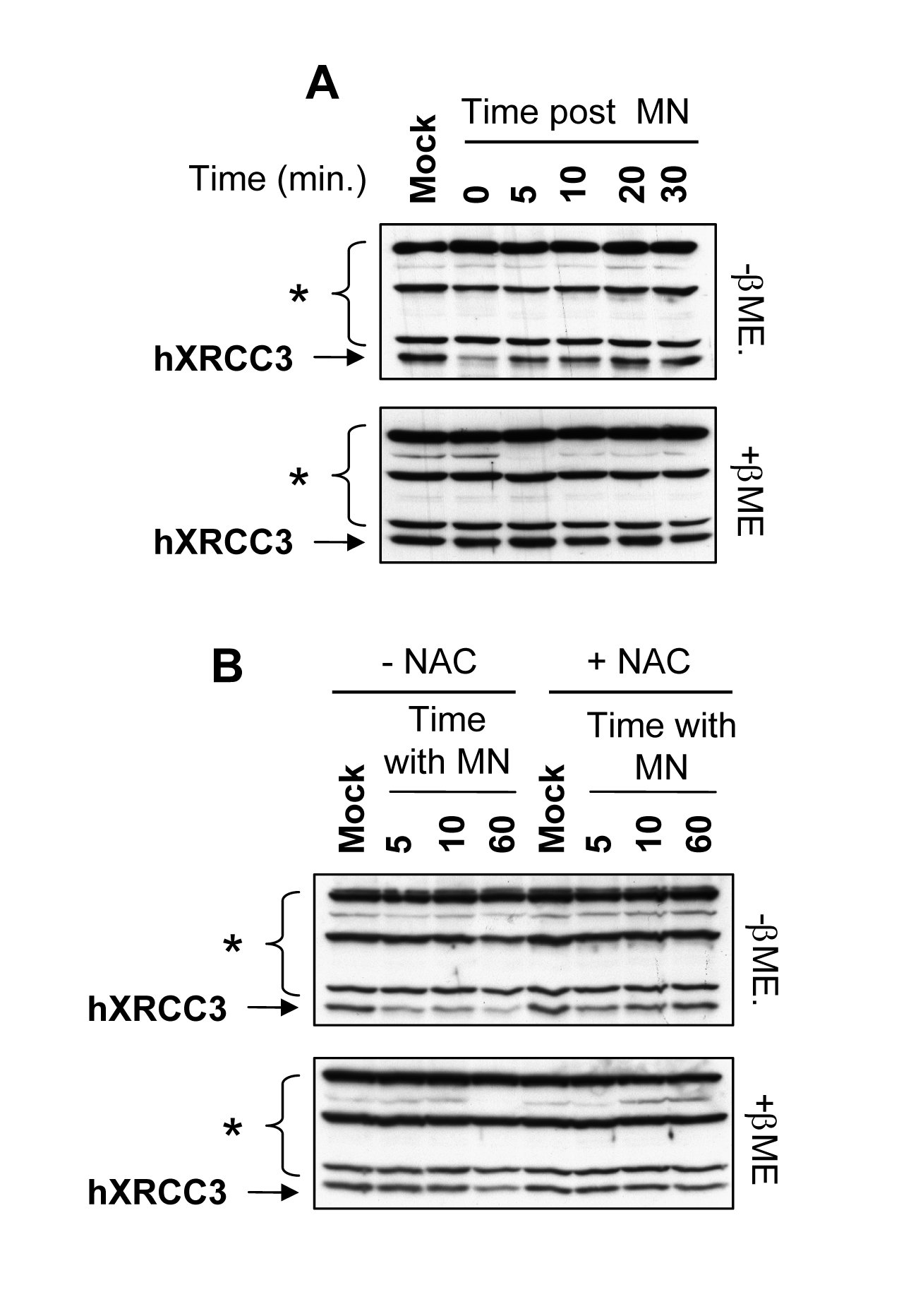

Supplement: Figure S9 — MN induces hXRCC3 oxidation in MRC5Vi cells. MRC5Vi cells were either incubated for 10 min (A) or for the indicated periods of time (B) with 100 µM MN in the presence or not of 10 mM NAC. Thereafter, the cells were lysed in buffer containing 10 mM NEM and the samples analysed by Western blot in non reducing (− ßME) or reducing (+ ßME) conditions. The star (*) indicates non-specific cross reactivity of the antibody. (TIF) [file pone.0075751.s009.tif]
